# Mesocrystals for photocatalysis: a comprehensive review on synthesis engineering and functional modifications

**DOI:** 10.1039/c8na00196k

**Published:** 2018-09-17

**Authors:** Shaodong Sun, Xiaojing Yu, Qing Yang, Zhimao Yang, Shuhua Liang

**Affiliations:** Shaanxi Province Key Laboratory for Electrical Materials and Infiltration Technology, School of Materials Science and Engineering, Xi'an University of Technology Xi'an 710048 Shaanxi People's Republic of China sdsun@xaut.edu.cn yxj@xaut.edu.cn liangsh@xaut.edu.cn; School of Science, State Key Laboratory for Mechanical Behavior of Materials, MOE Key Laboratory for Non-Equilibrium Synthesis and Modulation of Condensed Matter, Xi'an Jiaotong University Xi'an 710049 Shaanxi People's Republic of China

## Abstract

Mesocrystals are a new class of superstructures that are generally made of crystallographically highly ordered nanoparticles and could function as intermediates in a non-classical particle-mediated aggregation process. In the past decades, extensive research interest has been focused on the structural and morphogenetic aspects, as well as the growth mechanisms, of mesocrystals. Unique physicochemical properties including high surface area and ordered porosity provide new opportunities for potential applications. In particular, the oriented interfaces in mesocrystals are considered to be beneficial for effective photogenerated charge transfer, which is a promising photocatalytic candidate for promoting charge carrier separation. Only recently, remarkable advances have been reported with a special focus on TiO_2_ mesocrystal photocatalysts. However, there is still no comprehensive overview on various mesocrystal photocatalysts and their functional modifications. In this review, different kinds of mesocrystal photocatalysts, such as TiO_2_ (anatase), TiO_2_ (rutile), ZnO, CuO, Ta_2_O_5_, BiVO_4_, BaZrO_3_, SrTiO_3_, NaTaO_3_, Nb_3_O_7_(OH), In_2_O_3−*x*_(OH)_*y*_, and AgIn(WO_4_)_2_, are highlighted based on the synthesis engineering, functional modifications (including hybridization and doping), and typical structure-related photocatalytic mechanisms. Several current challenges and crucial issues of mesocrystal-based photocatalysts that need to be addressed in future studies are also given.

## Abstract 2

In this review, we mainly summarize the important progresses made in the development of photocatalysis-oriented mesocrystals, including synthesis engineering, functional modifications (hybridization and doping), and typical structure-related photocatalytic mechanisms. Some urgent issues and perspectives have also been discussed.

## Graphical abstract

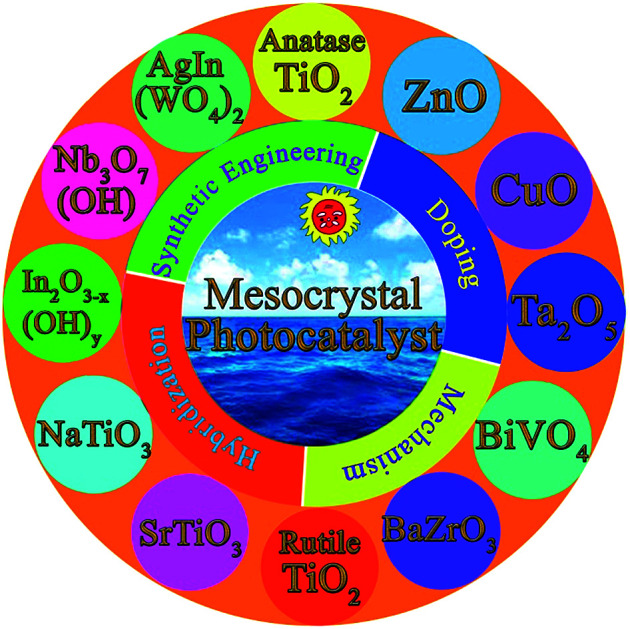

## 1. Introduction

Photocatalysis is a promising pathway to resolve the problems of energy depletion and environmental pollution *via* the photocatalytic conversion of solar light into chemical energy.^1–4^ Currently, the low utilization of visible light and short lifetimes of photogenerated charge carriers are two bottlenecks that limit the practical applications of photocatalysts.^5–7^ Therefore, improvements in the visible-light response and effective separation of photogenerated charge carriers for photocatalysts have garnered increased attention. Generally, the enhanced performances for single-component photocatalytic materials can be achieved by morphology-control or facet-control strategies. For example, a surface heterojunction can be constructed within a single anatase TiO_2_ polyhedron with coexposed (001) and (101) facets, which facilitates the transfer of photogenerated electrons and holes on the (101) and (001) facets, respectively, yielding better activity through the optimal ratio of the exposed (101) and (001) facets.^8^ However, the recombination of photogenerated charge carriers cannot be effectively restrained due to the lack of an energy barrier for charge separation, and mass migration is only occurring on the surfaces.^1^ Therefore, it is imperative to reasonably design and synthesize novel architectures for efficient interparticle or interfacial charge transfer, which can provide a significant breakthrough in optimizing single-component photocatalytic materials.

Mesocrystals are a new class of superstructures, which are generally made of crystallographically highly ordered nanoparticles and could function as intermediates in a nonclassical particle-mediated aggregation process.^9–13^ It should be noted that a mesocrystal is the definition of a superstructure and not a formation mechanism, which displays a selected area electron diffraction (SAED) pattern similar to a single crystal or a quasi-single crystal, resulting in the enhancement of charge migration than that in traditional polycrystalline materials. Moreover, as compared to conventional single crystals, mesocrystals possess nanoscale subunits, anisotropic shapes, rough surfaces, or order porosity, which exhibit high specific surface areas and offer more active sites for photocatalytic reactions. Consequently, these features provide new opportunities for potential photocatalytic applications.

In the past decades, extensive research interest has been focused on the structural and morphogenetic aspects, as well as the growth mechanisms of mesocrystals. A number of reviews on the formation mechanisms have been published, mainly including nanoparticle-oriented aggregation along an ordered organic matrix by spatial constraints and the assistance of external physical fields (such as electronic, magnetic, and optical fields).^14–25^ In 2016, remarkable advances were reported, particularly focusing on the use of TiO_2_ mesocrystal photocatalysts.^26^ However, there is still no comprehensive overview on the photocatalytic applications of various metal oxide mesocrystals and their functional modification forms so far.

**Scheme 1:**
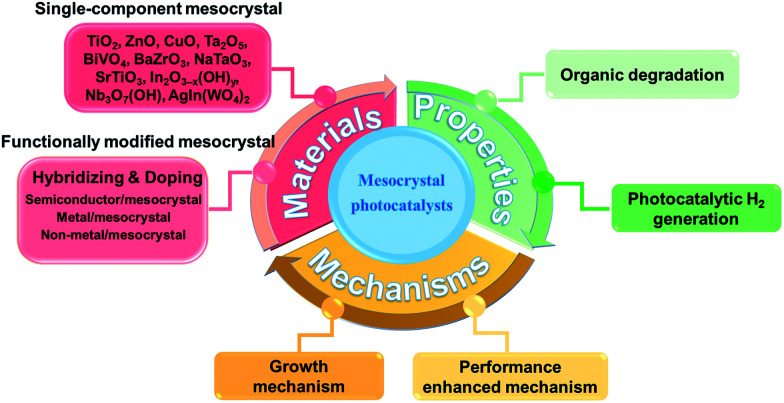
Summary of advancements in mesocrystal photocatalysts based on some basic aspects. The present review highlights significant advancements in diversified mesocrystal photocatalysts, including synthesis strategies leading to the growth of morphological mesocrystal micro/nanostructures, fundamental properties, and their current applications in the fields of degradation of organic pollutants and water splitting.

In this review, we mainly summarize the important progresses made in the development of photocatalysis-oriented mesocrystals in various species, including single-metallic oxides (such as TiO_2_ (anatase), TiO_2_ (rutile), ZnO, CuO, Ta_2_O_5_) and multimetallic oxides (such as BiVO_4_, BaZrO_3_, SrTiO_3_, NaTaO_3_, Nb_3_O_7_(OH), In_2_O_3−*x*_(OH)_*y*_, and AgIn(WO_4_)_2_) (Scheme 1). We start with the systematic review of the synthesis strategies and principles for enhanced performances of different mesocrystal photocatalysts. Next, the functional modifications (including hybridization and doping) for the construction of mesocrystal-based photocatalysts and structure-related photocatalytic mechanisms are presented. Finally, the current challenges and the crucial issues of mesocrystal-based photocatalysts that need to be addressed in future studies are mentioned.

## 2. Mesocrystal photocatalysts

Although a series of semiconductors with good structure–performance relationships have been investigated for improving the low quantum efficiency in photocatalysis, the existence of charge recombinations at the surface or inside the volume cannot be effectively avoided. With the development of a new class of porous metal oxide materials with uses ranging from catalysis to energy storage and conversion, highly ordered mesocrystal superstructures could limit the recombination of electron–hole pairs due to the anisotropic electron flow along the interparticles; therefore, the fast and efficient collection of photogenerated charge carriers on catalytic sites can be achieved by the dominant surface of mesocrystals. Therefore, understanding the synthesis strategies and corresponding formation mechanisms of typical single-component mesocrystal photocatalysts is significant for the controllable design of novel versatile mesocrystal-based composites. In this section, we mainly introduce the synthesis methods and photocatalytic mechanisms involving mesocrystal photocatalysts (Table 1), including TiO_2_ (anatase), TiO_2_ (rutile), ZnO, CuO, Ta_2_O_5_, BiVO_4_, BaZrO_3_, SrTiO_3_, NaTaO_3_, Nb_3_O_7_(OH), In_2_O_3−*x*_(OH)_*y*_, and AgIn(WO_4_)_2_. Initially, certain concepts involving the growth and formation mechanism of mesocrystals will be listed and explained. Topotactic transformation is a kind of structural transition in solid materials that enables structural transitions without losing the crystalline symmetry of the parental phase.^27,28^ Kirkendall effect is a kind of manifestation that induces void structures; due to the different diffusion rates between two species at an elevated temperature, movement can be induced at the interface between a diffusion couple.^29,30^ Oriented attachment is a nonclassical crystal growth model, where the nanocrystals align along a certain crystallographic direction in order to minimize their interfacial energy.^31,32^ Ostwald ripening process is a direct matter-relocation approach, which is defined as the “dissolution of small crystals or sol particles and the redeposition of the dissolved species on the surfaces of larger crystals or sol particles.”^33,34^

**Table 1:** Mesocrystal photocatalysts and their properties

Photocatalyst	Morphology	Synthesis process	Formation mechanism	Application	Mean reasons of enhanced property	Ref.
Anatase TiO_2_	Polyhedral mainly with exposed (001) facets	Prepared NH_4_TiOF_3_ mesocrystals and sintered at 700–900 °C	Topotactic transformation	Photodegradation of methylene blue	Special facets lead to a relatively large crystallite size	35
Anatase TiO_2_	Polyhedral mainly with exposed (001) facets from top view and (101) facets from side view	Prepared NH_4_TiOF_3_ mesocrystals and calcined at 500 °C	Topotactic transformation	Photocatalytic degradation of 4-chlorophenol and Cr^6+^ and photocatalytic hydrogen evolution	(1) (001) facets have strong ability to form hydroxyl radicals	37
					(2) Well-aligned nanocrystals can efficiently promote photoactive efficiency due to the facilitation of charge separation	
Anatase TiO_2_	Polyhedral mainly with exposed (001) facets	Sintered NH_4_TiOF_3_ precursors at 700–900 °C	Topotactic transformation	Photodegradation of methylene blue	Largely exposed (001) facets improved photodegradation	38
Anatase TiO_2_	Truncated tetragonal bipyramidal Wulff shape	Solvothermal synthesis: TiCl_4_ added into octyl alcohol and heated at 100 °C	Oriented attachment mechanism	Photodegradation of rhodamine B	A higher number of activity sites in this photocatalytic reaction improves photodegradation	39
Anatase TiO_2_	Sheet-like TiO_2_ mesocrystals with controllable nanothorns on the (101) facet	Intermediate NH_4_TiOF_3_ sheets treated with H_3_BO_3_ and NaOH, and then annealed at 500 °C	Topotactic transformation	Photocatalytic hydrogen evolution	Facet-induced charge separation and anisotropic electron flow improve photocatalytic property	40
Anatase TiO_2_	Layered nanosheets with exposed (001) facets	Hydrothermal method: using (NH_4_)_2_TiF_6_, H_3_BO_3_, and 2-propanol, followed by calcination treatment	Topotactic transformation	Photodegradation of rhodamine B	(001) facets provide highly active sites and layered structures facilitate the transport of reactants and degradation products	41
Anatase TiO_2_	Submicron-sized anatase TiO_2_ mesocrystals with exposed (001) surfaces	Solvothermal synthesis: NH_4_F and Ti(OC_4_H_9_)_4_ added into glacial acetic acid and then heated at 210 °C	Slow hydrolysis reaction controlled by the reaction between glacial acetic acid and Ti(OC_4_H_9_)_4_	Photodegradation of gaseous styrene	Large surface area, good anatase crystallinity, high percentage of (001) facets, wider band energy, and unique mesoporous structure combined together to improve photocatalytic property	42
Anatase TiO_2_	Regular-shaped TiO_2_ MCs enclosed with different proportions of (001) and (101) facets	Solvothermal synthesis: formic acid (FA) and titanium isopropoxide (TTIP)	Assembly of the subunits in ∼30–50 nm: FA molecules should be preferentially attached onto the specific (101) surfaces of the nanosheets, and therefore, lead to strongly anisotropic mutual interactions between formed small subunits	Photo-oxidation of nitrosobenzene	The synergistic effect of Ti^3+^, the higher proportion of (101) facets, and structural integrity of crystal are responsible for the higher photocatalytic activity	43
Anatase TiO_2_	Nanoporous spindles	Solvothermal synthesis: tetrabutyl titanate (TBT) was added dropwise to acetic acid (HAc) and maintained at 200 °C for 24 h	Hydrolysis reaction, controlled by the reaction between acetic acid and TBT, leading to the nanoporous structure and nano-size of TiO_2_	Photodegradation of gaseous benzene	The active anatase crystal phase, small crystallite size, high surface area, and narrow pore size distribution are important for yielding the best catalytic activity	46
Anatase TiO_2_	Spindles	Hydrothermal method + calcination: TBOT added into HAc solution with some water and then calcinated at different temperatures for 1 h	Hydrolysis reaction formed TiO_2_ and calcination temperature determined the structure of TiO_2_	Photodegradation of methylene blue	The TiO_2_ calcinated at a suitable temperature had increased the crystallinity and surface area, which were mainly responsible for the improvement in the photocatalytic properties	47
Anatase TiO_2_	Spindles	Solvothermal synthesis: TiCl_4_ aqueous solution was mixed with 40 mL CH_3_COOH and heated at 200 °C	Nanoparticle-oriented assembly	Photodegradation of methylene blue	The high orientation of primary nanocrystals in mesoporous structure accelerated efficient charge separation	48
Anatase TiO_2_	3D olive shape with subunits in spindles structure	After preferential evaporation of tetrahydrofuran (THF) solvent and TiO_2_ NPs assembled by PEO-PPO-PEO/titania oligomer spherical micelles are formed at the liquid−liquid interface	Evaporation-driven oriented assembly	Photocatalytic decomposition of methylene blue	Large number of oxygen vacancies located on the surface and high percentage of reactive (001) facets	49
Anatase TiO_2_	Hierarchical hollow microspheres	Ultrasound-assisted aerosol-spray method to prepare NH_4_TiOF_3_, and then, calcination for 2 h	Self-assembly and topotactic transformations	Photodegradation of 4-chlorophenol	Assembled nanosheets are favorable for multiple reflections, which greatly improve the utilization of UV light	52
Anatase TiO_2_	Porous microspheres	Solvothermal synthesis: TiOSO_4_ mixed with *tert*-butyl alcohol (molar ratio = 1 : 165), heated at 110 °C and annealed at 700 °C	Annealed temperature determined the orientation of mesoporous TiO_2_	Photodegradation of phenol and hydrogen evolution	Orderly arrangement of TiO_2_ nanocrystals can be more effective in the migration of photogenerated charges to have a higher photocurrent	53
Anatase TiO_2_	Nano-spherical assemblies (<100 nm)	Microemulsion method	Self-assembly and slow hydrolysis process	Photodegradation of methylene blue	Not mentioned	54
Anatase TiO_2_	Nano-spherical assemblies (<25 nm)	Microemulsion method	Using soft templates to assemble	Photodegradation of 2,4-dichlorophenol	High surface area and good crystallinity are effective for photodegradation	55
Rutile TiO_2_	Hierarchical assemblies	Microwave-assisted hydrothermal method	Self-assembly of small rutile TiO_2_	Photocatalytic oxidation of NO gas	A large effective surface area enabled the diffusive transport of photogenerated holes to oxidizable species	56
Rutile TiO_2_	Hollow microspheres	K_2_TiO_2_(C_2_O_4_)_2_ and HNO_3_ were added to H_2_O_2_ aqueous solution and then maintained at 80 °C	Hydrolysis-dissolution-precipitation	Photodegradation of rhodamine B	Mesopores in the mesocrystals contributed to absorb molecular dyes	57
Anatase TiO_2_	Sheets with exposed (001) facets	Microwave-assisted sonochemical method	Synergistic effect of microwave and sonication influence the hydrolysis process of TiO_2_ to induce oriented aggregation of TiO_2_ along the crystallographic axes	Photodegradation of rhodamine B	(1) Anatase mesocrystals have high energy facets, crystallinity	58
					(2) Mesocrystal structures are conducive to reduce electron–hole recombination rates	
Rutile TiO_2_	Nanosheets	Substrates were left to stand in TiCl_3_ aqueous solution in a polypropylene vessel and maintained at 25 °C for several days	Oxidative deposition	Photodegradation of methylene blue	Exposed crystal facets are beneficial toward electron transfer	59
Rutile TiO_2_	Porous sheets	Silica template hydrothermal method	Hydrolysis; HCl acted as etching agent	Photodegradation of methyl orange and hydrogen evolution	Effective reduction sites provided by the abundantly exposed (110) facets of rutile TiO_2_ improved the properties	61
Anatase TiO_2_	Porous sheets	Silica template hydrothermal method	Hydrolysis; HCl and HF acted as etching agents	Photodegradation of methyl orange and hydrogen evolution	The preferentially exposed (001) facets of anatase TiO_2_ are responsible for the high oxidative photocatalytic activity	61
Anatase TiO_2_	Rods	Solvothermal synthesis: Ti(OC_4_H9)_4_, CH_3_COOH, C_6_H_5_COOH, and CH_3_CH_2_OH were mixed and treated at 180 °C for 12 h	Hydrolysis; benzoic acid helped to form rod-like structures; alcohol and acetic acid helped to form mesoporous structures	Photodegradation of methyl orange	High crystallinity and higher specific surface area of the sample lead to more active sites and better adsorptive capacity	65
ZnO	Nanosheet assemblies	Zn(NO_3_)_2_ mixed with NH_4_F and NaOH and stirred at room temperature	The small units were prepared by chemical precipitation; then, the units formed mesoporous structures through epitaxial self-assembly	Photodegradation of methylene blue	Exposed nonpolar (10-10) facets of ZnO have higher photocatalytic activity	71
ZnO	Hollow assemblies	Hydrothermal synthesis: Zn(AC)_2_, HF, and hexamethylenetetramine mixture maintained at 160 °C for 6 h and calcined in air at 500/800 °C for 2 h	The Zn(OH)F precursor formed during the hydrothermal process and helped to increase the oxygen vacancies of ZnO	Photodegradation of methylene blue	Abundant oxygen vacancies play a key role in narrowing the bandgap instead of the formation of active centers or trap centers	73
ZnO	Nanosheet assemblies	Hydrothermal synthesis: PVP, Zn(NO_3_)_2_, and urea mixture maintained at 150 °C and calcined at 300–700 °C	PVP worked as structure-directing reagent to assist ZnO self-assembly	Photodegradation of methylene blue	The high-ordered MC structure can promote the separation and transfer of photoinduced electrons and holes and provided larger specific surface area	74
ZnO	Nanosheet assemblies	Hydrothermal synthesis: Zinc acetate solution and sodium citrate mixture heated to 150 °C for 24 h	Ostwald ripening process and nonclassical mesocrystal growth mechanism	Photodegradation of methylene blue and 2,4,6-trichlorophenol	(1) The ordered alignment of nanoparticles facilitated the transfer of photoinduced carriers	75
					(2) The defects located at the interfaces among the nanocrystals can act as active sites for photoreaction	
ZnO	Nanosheets	Electrodeposition: used ZnCl_2_, H_2_O_2_, and NaNO_3_ as the electrolyte composition, Al substrate as the working electrode and Pt foil as the counter electrode. The system worked under supercritical CO_2_ (SC–CO_2_) atmosphere	Oriented attachment describes a spontaneous self-assembly process to form mesocrystals. Meanwhile, Cl^−^ adsorption help to form 2D platelet structures. SC-CO_2_ helped to generate isotropical shape	Photoelectrochemical application	Highly oriented crystallinity and substantially long exciton lifetime of prepared ZnO improved photoelectrochemical properties	76
ZnO	Bundles	Precipitation-annealing: simply mixing the aqueous solutions of zinc acetate, sodium hydroxide, and tartaric acid and annealed at certain temperatures	Tartaric acid leads to oriented attachment of Zn(OH)_2_ and helps to assemble Zn (OH)_2_ bundles	Photodegradation of methyl orange and photoreduction of Cr^6+^ in water	The prepared catalysts had proper ZnO particle size and suitable porous structure	79
ZnO	Spindles	Ionic-liquid-based antisolvent method: ZnO-containing deep eutectic solvents was injected into (HOCH_2_)_3_CNH_2_ solution for 5 min at 70 °C	Tris molecules and deep eutectic solvents lead to oriented attachment and assembly of ZnO NPs	Photodegradation of methylene blue	(1) Mesostructure provided specific surface area and pore volume to improve photodegradation	81
					(2) C-planes of ZnO, worked as a superior facet for photodegradation, exposed on the surface of the mesocrystals	
CuO	Spindles	Additive-free complex-precursor solution method: Cu(NO_3_)_2_ solution mixed with NaOH solution stirred at 80 °C for 30 min	Hydrolysis–dissolution–precipitation and bottom-up assembly	Photodegradation of rhodamine B	These 3D mesostructural spindles exhibited more pores to absorb molecules and had the ability to reduce electron–hole recombinations	86
Ta_2_O_5_	Nanosheets	(NH_4_)_2_Ta_2_O_3_F_6_ nanorods were prepared by vapor hydrolysis reaction method. Then, as-prepared mesocrystalline nanorods were annealed at ∼700–900 °C for 3 h	Self-assembly of (NH_4_)_2_Ta_2_O_3_F_6_ and topotactic transformation	Photocatalytic hydrogen evolution	Mesocrystalline Ta_2_O_5_ superstructures contributed to the generation of long lifetime photoinduced carriers and effective conductive pathways for photocatalytic hydrogen production	88
BiVO_4_	Nanoparticle assemblies	Hydrothermal method: silica solution template filled with acidified BiVO_4_ precursor solution. Then, NaOH solution was used to form mesostructured BiVO_4_	BiVO_4_ nuclei grew by Ostwald ripening mechanism. Vacuum atmosphere is necessary to ensure sufficient infiltration into silica template.	Photocatalytic oxygen evolution	(1) Inner pores can scatter more light	95
					(2) High crystallinity and single coherent atomic configuration are good for the transfer of charge carriers	
					(3) Mesoporous structure can decrease the transfer distance	
					(4) Increase in surface area can also increase the active sites	
NaTaO_3_	Cubic assemblies	Surfactant-free solvothermal synthesis: TaCl_5_ in ethanol mixed with sodium ethoxide, heated at 240 °C for 4 h	Acidic alkoxide hydrolyzation yields particles with small size and high surface area	Photocatalytic hydrogen and oxygen evolution	Small particle size and high surface area improved the charge separation, migration of photogenerated carriers, and benefited the surface chemical reaction of catalysts	101
SrTiO_3_	Cubic assemblies	Hydrothermal treatment: TiO_2_ mesocrystals in ethanol were added into Sr(OH)_2_ solution. Then, they were mixed with NaOH, polyethylene glycol solution, and water, and heated at 200 °C for ∼12–60 h	Topotactic transformation	Photocatalytic hydrogen and oxygen evolution	Well-defined superstructure can deliver photo-charges more efficiently	105
SrTiO_3_	Porous spheres with wormhole-like structure	Solvothermal synthesis: Ti(C_4_H_9_O)_4_ and ammonia mixed to obtain a precipitated Ti(OH)_4_ and then added in Sr(NO_3_)_2_, KOH, and PVA solution and heated at 200 °C for ∼0.5–2 h	PVA leads to oriented aggregation and assembly of SrTiO_3_	Photodegradation of rhodamine B	High-crystalline SrTiO_3_ mesoporous spheres with large pores and primary nanoparticles of optimum size are good for photocatalytic reaction	107
In_2_O_3−*x*_(OH)_*y*_	Porous rods	Precipitation + calcination: InCl_3_, H_2_O, and urea are used to prepare In(OH)_3_ nanorods. Then, calcinated at 250 °C for 6 h	Nanorods are preferentially oriented such that their body lengths are aligned parallel to the substrate surface	Photoreduction of CO_2_	(1) Rod structure catalysts are more effective for inter-nanocrystal charge transfer	108
					(2) charge transfer may occur between neighboring nanocrystals in In_2_O_3−*x*_(OH)_*y*_ nanorods, resulting in prolonged lifetimes, thereby improving the photocatalytic activity	
					(3) In_2_O_3−*x*_(OH)_*y*_ nanorods populated with surface hydroxyl groups and oxygen vacancies to improve photocatalytic properties	
Nb_3_O_7_(OH)	Cubes with nanorods subunits	One-step hydrothermal method: cubes NbCl_4_-THF complex was mixed with HCl and then heated at 200 °C	Ostwald ripening process happened during the wire formation. Self-assembly *via* oriented aggregation	Photodegradation of methylene blue, rhodamine B, and indigo carmine	The mesocrystals benefit from their large surface area, high crystallinity, and direct electron transport path	109
AgIn(WO_4_)_2_	Hierarchical rods	Microwave-assisted approach: AgNO_3_ and In(NO_3_)_3_ mixed with Na_2_WO_4_ heated to 180 °C by microwave irradiation for 20 min	Oriented-attachment process accompanying the Ostwald ripening process	Photodegradation of eosin Y, rhodamine B, and methyl orange	Not mentioned	110

### 2.1 Binary oxide mesocrystals

#### 2.1.1 TiO_2_ mesocrystals

Titanium dioxide (TiO_2_) is one of the most promising photocatalysts due to its strong redox ability, high chemical stability, low toxicity, and low cost. Among the existing mesocrystal photocatalysts, the development of TiO_2_ mesocrystals has been attracting increased attention; therefore, the appearance and morphology details of TiO_2_ mesocrystals are abundantly available as compared to others, including ellipsoidals, spheres, polyhedra, nanosheets, and nanorods.^35–43,46–49,52–59,61,65^ In this subsection, we initially summarize the synthesis strategies of the different abovementioned species. Thereafter, versatile applications in photocatalytic hydrogen evolution and organics degradation, as well as the corresponding structure-related photocatalytic mechanisms, are discussed by using typical examples.

#### Three-dimensional (3D) architectures

It is well known that both external morphology and internal structure of a photocatalyst are very significant with regard to photocatalytic activity. With regard to the widely investigated TiO_2_ photocatalysts, its large crystallite size facilitates electron–hole separation; a large specific surface area can provide more reaction sites and large exposure of highly active facets are conducive toward high reaction activity on the reaction sites. All the above three advantages are necessary to obtain a good photocatalytic ability.^35^ However, it is difficult for an integrated structure to satisfy all the above three requirements until the development of mesocrystal photocatalysts. In particular, hierarchical 3D TiO_2_ mesocrystals could not only improve the relationship between crystallite size and specific surface area, but also offer a large number of high active (001) facets.

##### Polyhedral TiO_2_ mesocrystals

**Fig. 1:**
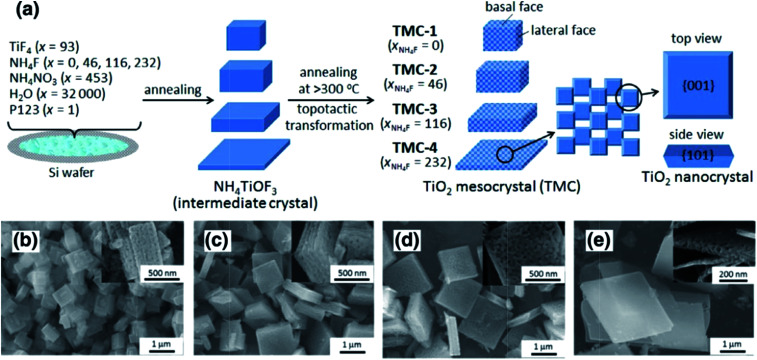
(a) Schematic illustration of the synthesis of differently shaped TiO_2_ mesocrystals. SEM images of different TiO_2_ mesocrystals prepared with *x* (molar ratio of NH_4_F) = 0 (b), 46 (c), 116 (d), and 232 (e). Insets display the corresponding high-magnification SEM images. It can be found that the thickness size of the as-synthesized particles gradually reduced along with an increase in the amount of NH_4_F. Adapted with permission from ref. 26. Copyright 2016 Elsevier.

The typical synthesis strategy for TiO_2_ mesocrystals comprising nanocrystals with exposed (001) facets involves the topotactic transformation of NH_4_TiOF_3_ mesocrystals at high annealing temperatures, which was first discovered by O'Brien's group in 2008.^36^ However, the experimental procedures were relatively complicated and could not be scaled up for practical applications. Therefore, it is necessary to develop a one-pot facile synthesis method to prepare TiO_2_ mesocrystals exposed with high active facets. Majima and coworkers have obtained plate-like TiO_2_ mesocrystals by annealing a thin layer of precursor solution containing TiF_4_, NH_4_F, and NH_4_NO_3_ on a silicon wafer. Evidently, the nonporous intermediate NH_4_TiOF_3_ precursor was first formed following the combination reactions from the mixtures of Ti^4+^, F^−^, NH^4+^, and H_2_O at low temperatures (<200 °C). As the annealing process required higher temperatures (>300 °C), the as-synthesized NH_4_TiOF_3_ precursor would be topotactically transformed into porous anatase TiO_2_ mesocrystals with dominant (001) facets. It should be noted that the gaps or holes are generated owing to nanocrystal diffusion and recrystallization during the removal of large amounts of N, H, and F atoms from the crystal lattice structure; therefore, the amount of NH_4_F is significant for the morphology-controlled synthesis of TiO_2_ mesocrystals (Fig. 1).^26^

The TiO_2_ mesocrystals assembled with highly ordered alignment of anatase nanocrystals (size: 40 nm) can display porous structures with pore diameters of several nanometers (surface area: >90 m^2^ g^−1^), resulting in remarkably long-lifetime charges, higher photoconductivity and photocatalytic hydrogen evolution, as well as degradation of 4-chlorophenol and Cr^6+^ in the aqueous phase.^37^ In this case, it was observed that TiO_2_ mesocrystals exposed with different facet ratios exhibited different reactivity orders during photooxidation, *i.e.*, (001) > (101), and photoreduction, *i.e.*, (101) > (001), under UV-light irradiation. Interestingly, the authors have confirmed that the (001) facets were preferable during molecular adsorption and photogenerated electron injection from the photoexcited dye sensitizers (eosin Y and Ruthenizer 470) to the conduction band (CB) of TiO_2_ under visible-light irradiation, whereas the (101) facets were beneficial for the collection of photogenerated electrons because of the directional electron flow. These findings emphasized that the concept of crystal-facet-dependent photocatalytic reactions can be extended to mesocrystal systems.

Based on the above synthesis route, Zhou and coworkers have developed TiO_2_ mesocrystals largely exposed with (001) facets through the topotactic annealing of NH_4_TiOF_3_ mesocrystals at 800 °C synthesized by (NH_4_)_2_TiF_6_ and NH_4_OH without the need for extremely toxic HF treatments.^35^ In this case, the specific structure of the TiO_2_ mesocrystal with a large crystallite size, high specific surface area, and additional numbers of active (001) facets can greatly enhance the photocatalytic activity, whereas lower or higher processing temperatures (*e.g.*, 700 °C and 900 °C, respectively) would damage the microstructure, and therefore, deteriorate the photocatalytic activity. In addition, these particles have good photochemical stability and much larger size than commercial P25, which suggests that they can be easily removed from the liquid-phase system by centrifugation and reused.^38^

Moreover, Leite and coworkers have proposed a kinetically controlled crystallization process by a nonaqueous sol–gel synthesis method to prepare anatase TiO_2_ recrystallized mesocrystals, a chemical process based on the reaction of titanium(iv) chloride with *n*-octanol. This is attributed to the oriented-attachment mechanism. During the oriented-attachment process, individual nanoparticles acting as primary building blocks could directly assemble with adjacent nanoparticles to yield a mesostructure with similar crystallographic orientation to minimize interfacial energy. The high-resolution transmission electron microscopy (HRTEM) analysis clearly revealed that the synthesized recrystallized anatase mesocrystals exhibited a truncated bipyramidal Wulff shape, indicating that its surface is dominated by (101) facets, which exhibited superior photoreactivity for rhodamine B degradation under visible-light irradiation as compared to commercial P25 as a benchmarking material.^39^

Furthermore, nanothorn TiO_2_ mesocrystals with dominant (101) facets displayed approximately 1.5 and 6 times higher photocatalytic hydrogen evolution activities than those of (001) facets and disordered nanocrystals, respectively, which can be attributed to the specific facet-induced charge separation and anisotropic electron flow. According to the single-particle photoluminescence measurements, Mott–Schottky analyses, and transient absorption measurements, efficient interfacial band alignment and charge separation were obtained, suggesting the contribution of the dominant (101) facet. This facet-induced anisotropic flow in the photocatalysis resulted in the realization of a photocatalyst that exhibited efficient charge separation and enhanced photocatalytic activity.^40^

**Fig. 2:**
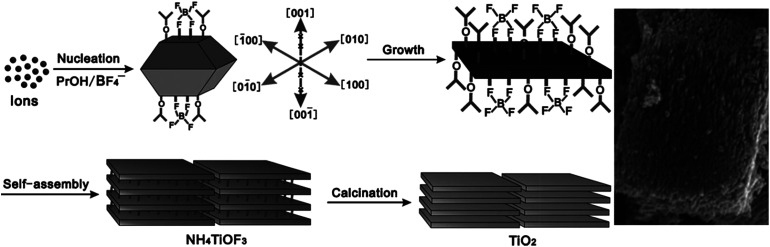
Schematic illustration of the formation of layered TiO_2_ mesocrystals. Adapted with permission from ref. 41. Copyright 2011 Wiley-VCH Verlag GmbH & Co.

In addition, layered TiO_2_ mesocrystals composed of nanosheets with exposed (001) facets were also successfully fabricated with the combination of hydrothermal treatments in the presence of (NH_4_)_2_TiF_6_, H_3_BO_3_, and PrOH, followed by calcination treatments. A schematic illustration of the formation of layered TiO_2_ is shown in Fig. 2.^41^ Initially, PrOH started to dissociate into PrO^−^ under weak acidic conditions; then, both PrO^−^ and BF_4_^−^ would preferentially bind to unsaturated Ti^4+^ cations on the (001) facets of NH_4_TiOF_3_ nuclei to reduce their surface energies, leading to the formation of single-crystal NH_4_TiOF_3_ nanosheets with (001) facets. Subsequently, these NH_4_TiOF_3_ nanosheets with (001) facets capped with PrO^−^ and BF_4_^−^ orderly assemble to block the tabular grains. Finally, NH_4_TiOF_3_ nanosheets were transformed into layered TiO_2_ nanosheets after calcination treatment because of the removal of PrOH and the reduced volume from NH_4_TiOF_3_ to TiO_2_. With regard to the photocatalytic activity, the as-prepared layered TiO_2_ with (001) facet nanosheets exhibited excellent performance for the degradation of rhodamine B when compared with commercial P25, which can be ascribed to the synergetic effects between the layered structures and (001) facets. Similarly, anatase TiO_2_ mesocrystals with exposed (001) facets have been successfully synthesized by a facile one-step solvothermal method using NH_4_F as the structure regulator in a glacial acetic acid environment, which can be used in the photocatalytic decomposition of gaseous styrene.^42^

It should be noted that the photocatalytic activity of polyhedral TiO_2_ mesocrystals with controllable proportions of (101) and (001) facets is significant, so it is highly desirable to investigate the photocatalytic activities of TiO_2_ mesocrystals with different (101)/(001) facet ratios. Fortunately, Zhao and coworkers have synthesized regular-shaped TiO_2_ mesocrystals enclosed with different proportions of (001) and (101) facets by a simple approach in the presence of formic acid and titanium isopropoxide as the original reactants without any other additives and surfactants at 160 °C. Further, the control of the (101)/(001) ratio of TiO_2_ mesocrystals was achieved by altering the solvothermal treatment periods.^43^ The TiO_2_ mesocrystals enclosed by a high proportion of (101) facets showed higher photocatalytic activity for benching nitrosobenzene than those with a lower proportion, which was attributed to the synergistic effect of Ti^3+^ and the proportion of (101) facets. In addition, the normalized photocatalytic activity of TiO_2_ mesocrystals was better than that of nanocrystals as the proportion of (101) facets was equal, suggesting that the structural integrity played a key role in the photocatalytic activity.

##### Spindle-shaped TiO_2_ mesocrystals

The formation of a spindle shape is common for metal oxide mesocrystals due to the oriented attachment of the nanoparticles. Based on earlier literature,^44–48^ it is found that spindle-shaped anatase TiO_2_ mesocrystals can be facilely synthesized on a large scale through mesoscale assembly in titanium salt/acetic acid systems without any additives under solvothermal conditions. The acetic acid and solvothermal conditions are the key factors for the synthesis of spindle-shaped TiO_2_ mesocrystals, whereas the type of titanium salt is adjustable, including tetrabutyl titanate,^44,45^ butyl titanate,^46^ titanium butoxide,^47^ and titanium tetrachloride.^48^

**Fig. 3:**
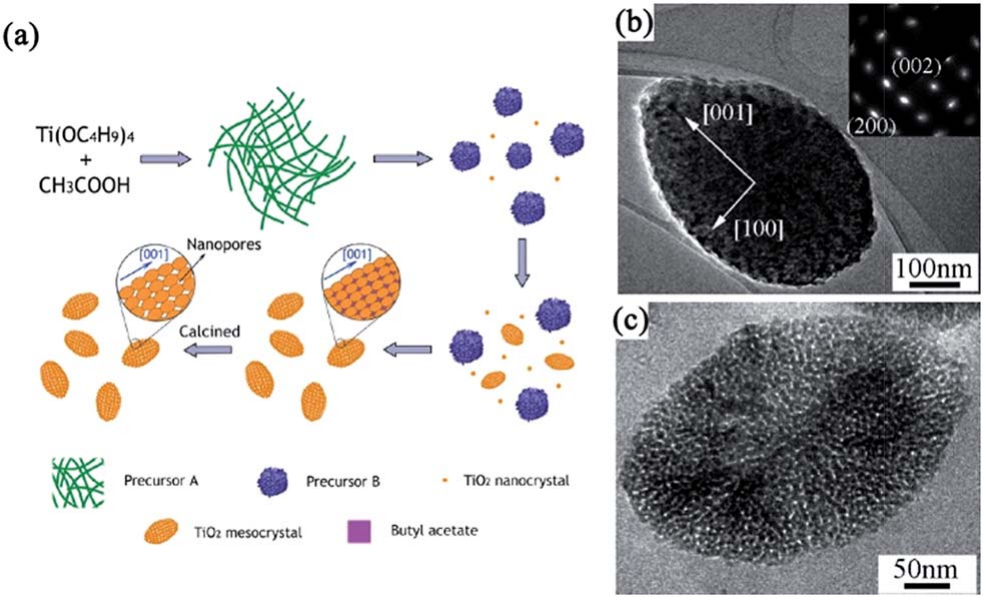
(a) Schematic illustration of a tentative mechanism for the additive-free synthesis of porous anatase TiO_2_ mesocrystals. (b) A low-magnification TEM image of a single spindle. Inset shows the corresponding SAED pattern. (c) A high-magnification TEM image of a porous particle. Adapted with permission from ref. 44. Copyright 2011 American Chemical Society.

For example, Qi and coworkers have demonstrated the first additive-free synthesis of porous anatase TiO_2_ mesocrystals with a single-crystal-like SAED pattern by using tetrabutyl titanate as the titanium source and acetic acid as the solvent.^44^ A complex nanoparticle-assembly process was obtained, involving the slow release of soluble species from metastable solid tetrabutyl titanate precursors for the continuous formation of anatase building blocks, followed by the oriented aggregation of tiny anatase nanocrystals under the capping of the as-produced butyl acetate, finally leading to the formation of porous spindle-shaped mesocrystals after the removal of organic residuals by calcination treatment. A schematic illustration of a tentative mechanism for the formation of porous anatase TiO_2_ mesocrystals without additives is shown in Fig. 3a. Fig. 3b shows a typical transmission electron microscopy (TEM) image of a single spindle particle, indicating that the particle consists of nanosized subunits. Its SAED pattern exhibits diffraction spots corresponding to the (010) zone axis of the anatase-phase TiO_2_, suggesting the possession of a “single-crystal-like” structure. Notably, it can be seen that the diffraction spots are slightly elongated, indicating that there is a small lattice mismatch between the boundaries of the nanoparticles, which is a typical characteristic of mesocrystals. The internal porosity of the particle is revealed by the TEM image taken on a microtomed sample (Fig. 3c). Lim and coworkers have synthesized spindle TiO_2_ mesocrystals using a hydrothermal method in the presence of titanium butoxide/acetic acid/water system and investigated the effect of calcination temperature (100–800 °C) on their morphology, crystallinity, and photocatalytic activity.^47^ In this work, the authors have found that controlling the calcination temperature is an effective pathway to control the morphology, crystallinity, and photocatalytic activity of TiO_2_ mesocrystals. The shape, dimension, and crystal structure of the TiO_2_ mesocrystals have no appreciable changes as the calcination temperature increased to 300 °C, and the crystallinity can be improved by increasing the temperature. However, the mesocrystal characteristic began to disappear at 400 °C, and the specific surface area decreased with increasing temperature due to the reduced boundaries. Hence, the photocatalytic degradation of methylene blue for TiO_2_ improved when the temperature increased to 300 °C owing to the enhanced crystallinity and elimination of byproducts; however, it became poor above 400 °C because of the decreased surface area.^47^ In addition, mesoporous anatase TiO_2_ prepared by using butyl titanate as the Ti source and acetic acid as the solvent can be used toward inducing photocatalytic activity in the degradation of gaseous benzene.^46^

Apart from the above solvothermal methods, the hard-template strategy has also been developed for the fabrication of porous TiO_2_ mesocrystals. Zhao and coworkers have reported a facile evaporation-driven oriented assembly strategy to prepare olive-shaped mesoporous TiO_2_ mesocrystals in an acidic tetrahydrofuran (THF)/pluronic F127/water/HCl/acetic acid/titanium tetrabutoxide mixed solution,^49^ which started with the liquid–liquid phase separation as the preferential evaporation of THF at 60 °C. Then, spindle-shaped TiO_2_ particles assembled by pluronic F127/titania oligomer spherical micelles were generated at the liquid–liquid interface. Finally, 3D-open anisotropic spindle-like mesoporous TiO_2_ mesocrystals were obtained by the continuous evaporation of residual THF and hydrolyzed solvents, which could drive the oriented attachment of both mesopore channels and flake-like nanocrystals from the initial spherical composite micelles along the free radial and restricted tangential directions. Dye-sensitized solar cells based on the above samples showed ultrahigh photoconversion efficiencies (beyond 11%), which were attributed to the intrinsic mesocrystal nature as well as high porosity.

##### Mesocrystalline TiO_2_ assemblies

**Fig. 4:**
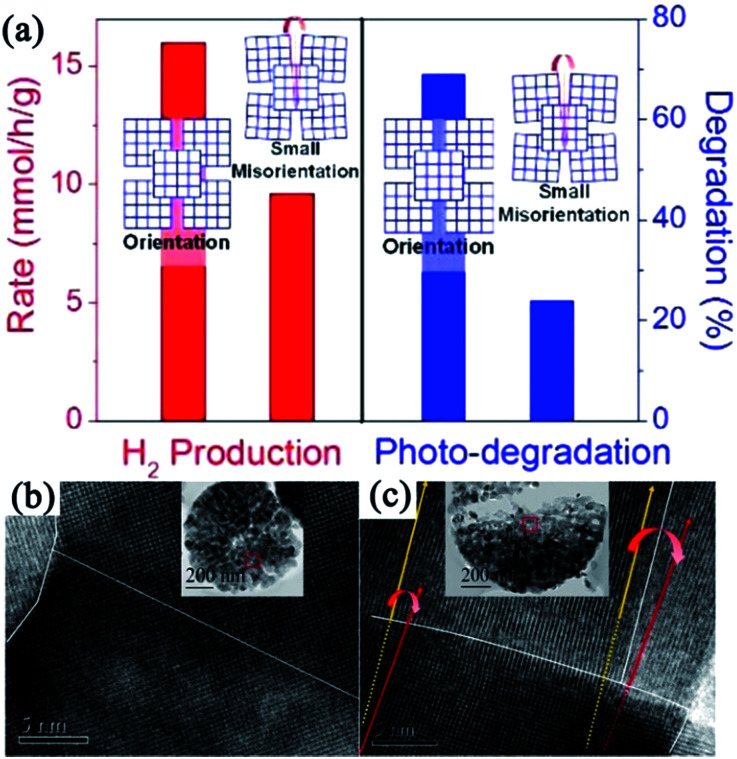
(a) Schematic illustration of disordered and ordered aggregations of TiO_2_ nanoparticles and their corresponding photocatalytic activities. (b) HRTEM image of TiO_2_ mesocrystal with ordered orientation. (c) HRTEM image of TiO_2_ mesocrystal with misorientation (crystal lattice mismatch). Adapted with permission from ref. 53. Copyright 2015 American Chemical Society.

Although the studies on TiO_2_ mesocrystal photocatalysts have been mainly concentrated on the structure/morphology and porosity control by the abovementioned different synthesis techniques, the deep understanding the relationship between the oriented-attachment fashion and photocatalytic efficiency has been rarely investigated. Bian and coworkers have observed that TiO_2_ mesocrystals showed largely enhanced photoconductivity and photocatalytic activities than those of polycrystalline materials because of the remarkably increased long-lifetime charges under illumination for TiO_2_ mesocrystals.^50,51^ They have also synthesized hierarchical hollow microspheres with TiO_2_ mesocrystal nanosheets as building blocks by an ultrasound-assisted aerosol-spray method followed by topotactic transformations. TiO_2_ mesocrystal hollow microspheres can largely enhance photocatalytic performances.^52^ Furthermore, they found, for the first time, that the presence of small misorientations had an obvious harmful effect on the charge transfer, and hence, largely suppressed the photocatalytic efficiencies, as shown in Fig. 4.^53^ The involved two typical TiO_2_ spherical mesocrystals, one with oriented and the other with misoriented alignment, of secondary nanocrystals were prepared from a precursor containing TiOSO_4_ and *tert*-butyl alcohol by thermal annealing and hydrothermal recrystallization processes, respectively. Therefore, this finding should be taken into account and avoided during the design and synthesis of semiconductor mesocrystal photocatalysts.^53^

It is well known that the size effect is also significant for photocatalytic activity; therefore, the preparation of sub-100 nm mesocrystal-like porous assemblies is imperative. Tartaj and coworkers have developed an inverse microemulsion method for the synthesis of TiO_2_ mesocrystalline assemblies with mesopores, and all the sizes ranged from 25 to 70 nm. These 25 nm nanostructures exhibited good electrochemical performance and good capability for photocatalytic degradation.^54,55^

From the abovementioned results, it can be found that anatase TiO_2_ mesocrystals can be synthesized by a facile process, whereas rutile TiO_2_ mesocrystals for photocatalysis have been rarely reported. Jimmy C. Yu and coworkers have reported a simple and environmentally friendly approach for preparing photocatalytically active rutile TiO_2_ mesocrystals by a microwave-assisted hydrothermal method involving titanium(iii) chloride as the only reactant. The as-synthesized one-dimensional (1D) rutile nanowires can easily assemble into three-dimensional (3D) hierarchical architectures without the help of surfactants or additives.^56^ Similarly, Wu and coworkers synthesized hollow TiO_2_ microspheres assembled with rutile mesocrystal nanorods directly from a mixed aqueous solution of K_2_TiO(C_2_O_4_)_2_, H_2_O_2_, and HNO_3_ at a low temperature of 80 °C, which displayed remarkable photocatalytic activity in photodegrading rhodamine B solution under UV-light illumination.^57^

#### Two-dimensional (2D) architectures

Thus far, the literature on 2D TiO_2_ mesocrystals is not exhaustive. Pillai and coworkers have demonstrated anatase TiO_2_ mesocrystals with exposed (001) facets and sheet-like morphologies by a facile halide-free low-temperature microwave-assisted sonochemical method, which exhibited good crystallinity, higher surface area, and excellent stability.^58^ In particular, sheet-like mesostructures played a crucial role in electron transport, which enabled faster charge transfer as confirmed by the enhanced photocurrent of 131 nA at 0 V bias. Therefore, this environmentally friendly synthesis strategy enabled 2D anatase mesocrystals to exhibit high energy facets, crystallinity, and low recombination rate of the photogenerated charge carriers, and therefore, resulted in improved photocatalytic activity. The effect of charge carriers on the photocatalytic activity was investigated in detail, and it followed the order: hydroxyl radicals > holes > electrons.^58^ Moreover, 2D rutile TiO_2_ mesocrystals can also be prepared by the deposition route in the presence of glass substrates, and a titanium source (TiCl_3_) facilitates this process at room temperature for several days. Rutile nanosheets with thickness of 10 nm and width of 200–600 nm consisted of oriented nanocrystals with size of 2–3 nm. The unit rutile nanocrystals were surrounded by a combination of (101) and (110) facets or (111) and (110) facets. In particular, a higher specific surface area was achieved on the rutile nanosheet with a mesocrystal interior, which displayed enhanced photocatalytic decomposition of an organic dye.^59^

**Fig. 5:**
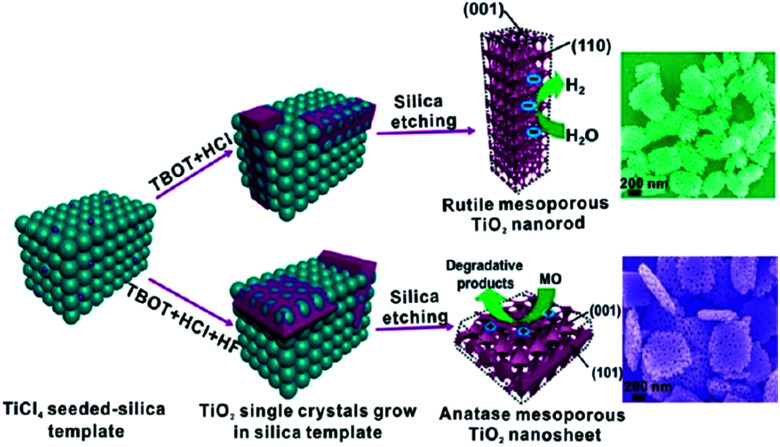
Schematic illustration of the synthesis pathways of rutile TiO_2_ mesocrystals and anatase TiO_2_ mesocrystals using silica templates by a hydrothermal method. Adapted with permission from ref. 61. Copyright 2013 American Chemical Society.

Using a hard template is an effective alternate strategy for the synthesis of porous TiO_2_ nanosheets, which involved heterogeneous crystal nucleation and oriented growth within the templates.^60^ Hence, a series of mesoporous single-crystal-like structures, including anatase mesoporous TiO_2_ nanosheets with dominant (001) facets and rutile mesoporous TiO_2_ nanorods with tunable sizes, have been obtained in the presence of silica, titanium tetrachloride, titanium butoxide, hydrochloric acid, and hydrofluoric acid (Fig. 5).^61^ The resultant mesoporous TiO_2_ single-like crystals displayed enhanced photocatalytic performances on hydrogen evolution and degradation of methyl orange owing to their enlarged surface area, single-crystal nature, and exposure of reactive crystal facets coupled with a 3D connected mesoporous architecture. It was observed that the (110) facets of rutile mesoporous TiO_2_ can be essentially considered as reductive sites in the photoreduction reaction, while the (001) facets of anatase mesoporous TiO_2_ exhibited oxidation sites in the oxidative process.^61^ However, the use of a strong acid is not ecofriendly, so it is still a challenge to develop a new hard-template technology that can be used to fabricate mesoporous TiO_2_ mesocrystals with tunable facets and crystalline phase.

##### 1D architectures

**Fig. 6:**
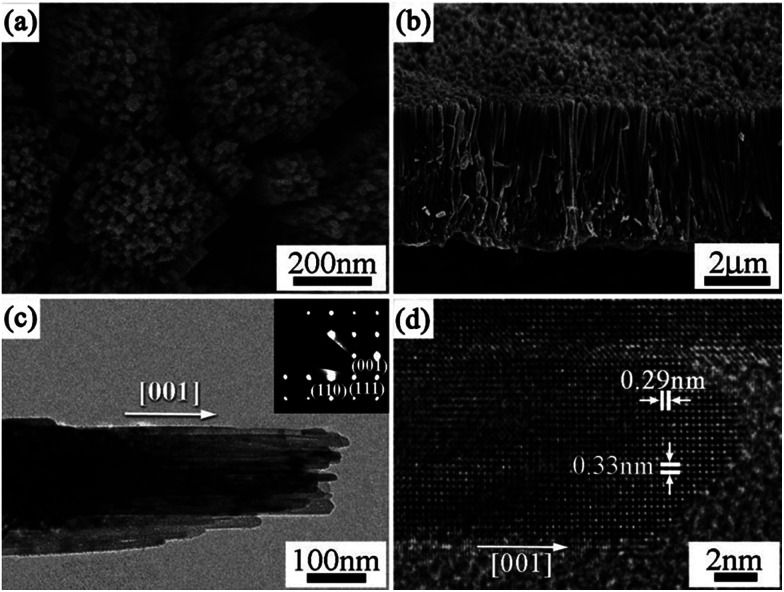
(a and b) SEM, (c) TEM, and (d) HRTEM images of rutile TiO_2_ nanorod arrays prepared at 150 °C on Ti foil for 20 h. The inset in (c) is the corresponding SAED pattern. Adapted with permission from ref. 64. Copyright 2012 Royal Society of Chemistry.

1D photocatalysts could trap solar light along their long axial direction and the simultaneous efficient carrier separation and collection in the nanometer-scale radial direction, resulting in the enhancement in the photocatalytic activity. Therefore, the synthesis of 1D TiO_2_ mesocrystals has attracted considerable attention.^62,63^ Qi and coworkers reported excellent broadband and quasi-omnidirectional antireflective structures based on highly stable, self-cleaning, mesocrystalline rutile TiO_2_ nanorod arrays (Fig. 6),^64^ which were prepared by a simple hydrothermal treatment of Ti foils in the presence of tetrabutyl titanate and hydrochloric acid. In this case, the nanorod building block is a single-crystal-like rutile TiO_2_ mesocrystal comprising many (001)-oriented nanotips (diameter: approximately 10–30 nm) grown on the top of a (001)-oriented stem nanorod (diameter: about 100–400 nm). The hierarchical TiO_2_ nanorod arrays showed the efficient suppression of reflection toward wavelengths ranging from visible to near-infrared (NIR) region, which was attributable to an optimized graded refractive index profile resulting from the multi-tips-on-rod structures.^64^ Liu and coworkers have synthesized rod-like TiO_2_ anatase mesocrystals with high specific surface area and excellent photocatalytic activity by a mild solvothermal route,^65^ in which the reagents were Ti(OC_4_H_9_)_4_, CH_3_COOH, C_6_H_5_COOH, and CH_3_CH_2_OH. It can be proposed that the oriented attachment of TiO_2_ nanoparticles was carried out under the synergism of hydrophobic bonds, π–π interactions, and mixed-ester templates. Further, the growth of the crystal facet of anatase was also affected by the π–π interactions. This study not only opens up new avenues for rationally designing TiO_2_ mesocrystal materials with ideal hierarchy and controllable sizes, but also provides a perspective toward uncovering the formation process of porous-crystalline superstructures.

Based on the abovementioned synthesis strategies, it can be observed that the species of precursors, reaction temperature, etching agent, and ratio of controlling agent play a significant role in the synthesis of novel TiO_2_ mesocrystals for photocatalysis. The improvement in the photocatalytic activity for TiO_2_ mesocrystals can be attributed to the synergistic effect of mesostructure (including size, morphology, and crystalline phase), (101)/(001) facet ratio, and Ti^3+^ vacancy. However, the integration process of the above parameters in a mesocrystal photocatalyst is still in its infancy. Therefore, it is necessary to develop a new strategy in this field.

#### 2.1.2 ZnO mesocrystals

Zinc oxide (ZnO) is a well-known wide bandgap semiconductor, which has important applications in photocatalyses.^66–68^ A number of ZnO mesocrystals have been successfully prepared, including nanosheet-assembled mesocrystals,^69–76^ microspheres,^77,78^ rod-like bundles,^79^ and spindles.^80,81^ However, ZnO mesocrystals for photocatalysis have not been studied so far. Here, the structural and morphogenetic aspects of ZnO mesocrystals are initially summarized, and their photocatalytic applications are simultaneously discussed.

##### Mesocrystalline ZnO assemblies

Thus far, the most common morphology of a ZnO mesocrystal is 3D hierarchical architecture with mesocrystalline ZnO building blocks. The ZnO lattice has both polar surfaces, (0001) as well as (0001̄), and a nonpolar surface (101̄0), which differently interact with the surface-protecting surfactants or polymers. Generally, it is facile to obtain ZnO nanoplates instead of nanorods because of the oriented attachment of ZnO nanoparticles with their nonpolar surfaces by protecting the polar surfaces, and mesocrystalline ZnO assembly with stacked nanoplate building blocks would be generated through the capping agent or intrinsic electrostatic field.^69^ Mou and coworkers have prepared a nacre-like hierarchical mesocrystal structure of ZnO in the presence of a mixture of gelatin/Zn(NO_3_)_2_·6H_2_O/hexamethylenetetramine, where biopolymer gelatin containing many polar amino acids act as the surface-protecting agent for the polar surfaces of ZnO, finally resulting in the formation of micrometer-sized ZnO mesocrystals with hexagonal shapes resulting from the stacked nanoplates.^69^ Similarly, Lee and coworkers have reported a facile, low-temperature synthesis approach in an aqueous solution for the synthesis of various ZnO mesocrystals (including platelets, rings, and ellipsoids) due to the oriented attachment of ZnO nanoparticles, where the surfactant cetyltrimethylammonium bromide (CTAB) played two critical roles, namely, shape control and micelles for the aggregation of nanoparticles with temperature changes.^70^ Typically, the samples were prepared by injecting an aqueous solution of ammonia into a Zn(NO_3_)_2_ solution in the presence of CTAB. CTAB-mediated zinc hydroxy double salt (zinc-HDS) mesocrystal sheets were synthesized at room temperature, and these Zn-HDS mesocrystal sheets can be decomposed into ZnO superstructure with rigid hexagonal morphology as the reaction temperature increased. Significantly, Wang and coworkers have demonstrated the fast and spontaneous room-temperature formation of ZnO mesocrystals constructed with nanosheet building blocks by the edge-sharing lateral attachment of 1D nanorods for the first time,^71^ which involved the phase transformation from two intermediate compounds, namely, ZnF(OH) and Zn(OH)_2_. The epitaxial attachment of ZnO (101̄0) nanosheets led to the assembly of hierarchical mesocrystals, which was confirmed to be a geometrically ideal photocatalyst that was easily separable and recyclable. The superior efficiency of the UV and visible photocatalytic degradation of methylene blue can be ascribed to the maximized exposure of the reactive (101̄0) facets in the epitaxially assembled superstructures.

Furthermore, a hydrothermal strategy is an effective alternative for the synthesis of mesocrystalline ZnO assembly.^72^ For example, Xu and coworkers have prepared stable yellow ZnO microring mesocrystals with a relatively narrow bandgap (*E*_g_ = 3.09 eV) and visible-light response by the hydrothermal route in the presence of hexamethylenetetramine/HF/zinc acetate dihydrate at 160 °C for 6 h.^73^ Raman and X-ray photoelectron spectroscopy spectra revealed that a large amount of oxygen vacancies existed in the yellow ZnO mesocrystals, resulting in the narrowing of the bandgap and an increase in the visible-light response of yellow ZnO. Further, the concentration of oxygen defects decreased with an increase in the annealing temperature in air. In addition, the electron paramagnetic resonance spectra confirmed that the yellow ZnO mesocrystals possessed abundant surface defects, leading to strong photoluminescence emission. Therefore, the yellow ZnO mesocrystals with highly ordered porous structures were found to be efficient for the photodecomposition of methyl blue under visible-light irradiation, which were favorable for directional transport and efficient charge carrier separation. It should be noted that these yellow ZnO microrings were very stable for at least one year. Moreover, various shaped ZnO architectures were synthesized through a simple hydrothermal route in the presence of a soft template as a structure-directing reagent.^74^ The flower-like hierarchical assembly was constructed with leaf-shaped mesocrystals that were composed of nanocrystals aligned along the (111) orientation, which displayed the highest photocatalytic activity when compared with the counterpart of nanocrystal ZnO, pencil-shaped mesocrystal ZnO, and plate-like mesocrystal ZnO. The improved photocatalytic activity could be attributed to not only the hierarchical structure, large specific surface area, and high crystallinity, but also the highly ordered mesostructured architecture.

Apart from the morphological architectures, defects engineering of photocatalysts is significant in the determining the photocatalytic activity. Wang and coworkers have reported that the interface-defect-mediated photocatalytic activity of pompon-like ZnO mesocrystal photocatalyst could be synthesized *via* a hydrothermal approach in the presence of sodium citrate without any other organic templates.^75^ The as-prepared pompon-like ZnO assemblies were composed of mesocrystal nanosheets with exposed high energy (002) facets having high crystallinity. Here the defects were located at the interfaces among the nanocrystals in the ZnO mesocrystals, playing a key role in the photocatalytic degradation of organic pollutants (such as methylene blue and 2,4,6-trichlorophenol) than that of interstitial zinc vacancies in bulk.

Electrodeposition can also be used to synthesize ZnO mesocrystals. Lin and coworkers first developed an effective supercritical CO_2_ (sc-CO_2_) emulsion-assisted electrochemical strategy for the cathodic deposition of ZnO mesocrystals.^76^ The deposition process involved the formation of primary nanocrystals possessing high surface energy, followed by the oriented attachment of primary nanocrystals along an energetically favorable orientation to generate ZnO mesocrystals. Because of the highly oriented crystallinity of mesocrystals, the as-deposited ZnO mesocrystals exhibited remarkable near-band-edge emissions at room temperature and substantially long exciton lifetime, which can limit the nonradiative charge recombination to extend the exciton decay dynamics. Hence, these ZnO mesocrystals exhibited largely enhanced photoactivity toward photoelectrochemical water oxidation, which was attributed to the advantageous structural characteristics of mesocrystals, including high crystallinity and abundant porosity.^76^

##### Mesocrystalline ZnO microspheres

**Fig. 7:**
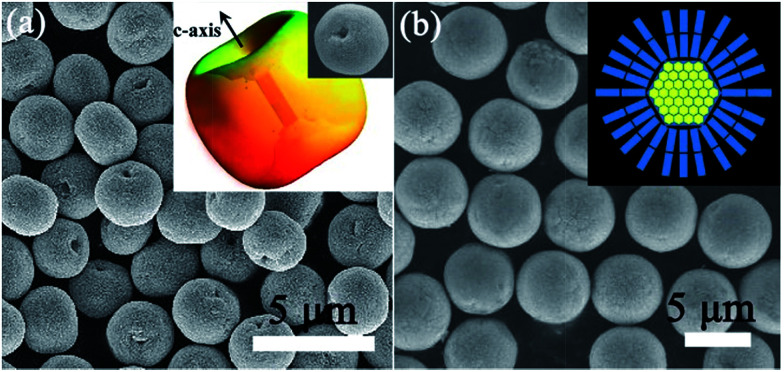
(a) SEM image of ZnO apple-like structures. Adapted with permission from ref. 78. Copyright 2011 Nature Publishing Group. Inset shows the corresponding schematic illustration and a typical particle. Adapted with permission from ref. 77. Copyright 2009 American Chemical Society. (b) SEM images of the ZnO mesocrystal microspheres. Inset is the corresponding schematic illustration. Adapted with permission from ref. 78. Copyright 2011 Nature Publishing Group.

As mentioned above, the dipole-induced electrostatic interactions between the building units can act as the aligning force for the formation of ZnO mesocrystals. However, the dipole-field-induced assembly along the *c*-axis forming anisotropic ZnO superstructures has been less investigated. Liu and coworkers have demonstrated the direct evidences for a unique core–shell-structured ZnO mesocrystal microsphere constructed by densely packed nanoplatelets by a low-temperature, polymer-mediated, one-pot hydrothermal route in the presence of a water-soluble polymer poly(sodium-4-styrenesulfonate).^77^ These nanoplatelet-based core–shell mesocrystal ZnO microsphere formed *via* a nonclassical crystallization process, which involved the synergistic effects of the electric fields of the core and the dipole–dipole interaction between the nanoplatelets on the shell. The calculation based on a dipole model confirms the dipole-field-driven mechanism forming apple-like structures and mesocrystals, as shown in Fig. 7.^77^ Significantly, green light can stimulate terahertz emissions from these core–shell mesocrystal ZnO microspheres.^78^

##### Mesocrystalline ZnO bundles

**Fig. 8:**
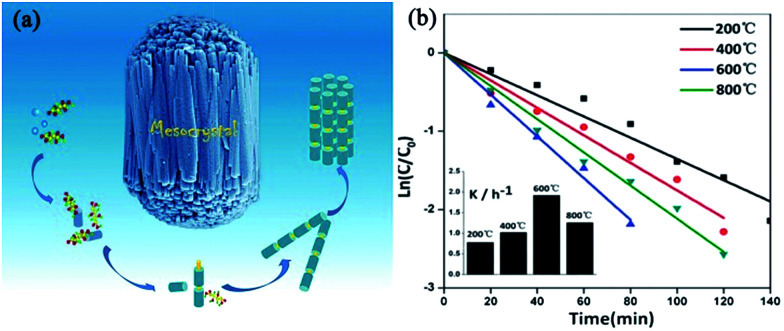
(a) Schematic illustration of the growth pathways of bundle-like ZnO mesocrystals. (b) Photocatalytic dynamics curves of methyl orange with ZnO mesocrystals synthesized at 200, 400, 600, and 800 °C as catalysts. Adapted with permission from ref. 79. Copyright 2013 Royal Society of Chemistry.

Similar to the formation of TiO_2_ mesocrystals, the topotactic transformation of precursor mesocrystals at high annealing temperatures is also suitable for the synthesis of ZnO mesocrystals. For example, Guo and coworkers have reported a simple and scalable wet-chemical route combined with a facile post-annealing process to produce rod-like ZnO mesocrystals with l(+)-tartaric acid (TA) as the orientation inducer.^79^ In this synthesis process, the authors proposed that the mild acidic characteristics and unique molecular structure of TA is important in assembling Zn(OH)_2_–TA into having unique mesostructural morphology. Then, the mesocrystals composed of ZnO nanoparticles were generated after being annealed in air at certain temperatures, which inherited the rod-like morphology of Zn(OH)_2_–TA composites. A schematic representation of the formation mechanism of rod-like ZnO mesocrystals is shown in Fig. 8a. The annealing temperature played a crucial role in the photocatalytic performance, as shown in Fig. 8b. In comparison with individual ZnO nanoparticles, ZnO mesocrystals exhibited decent photocatalytic activities with respect to the photodegradation of methyl orange and photoreduction of Cr^6+^.

##### Mesocrystalline ZnO spindles

**Fig. 9:**
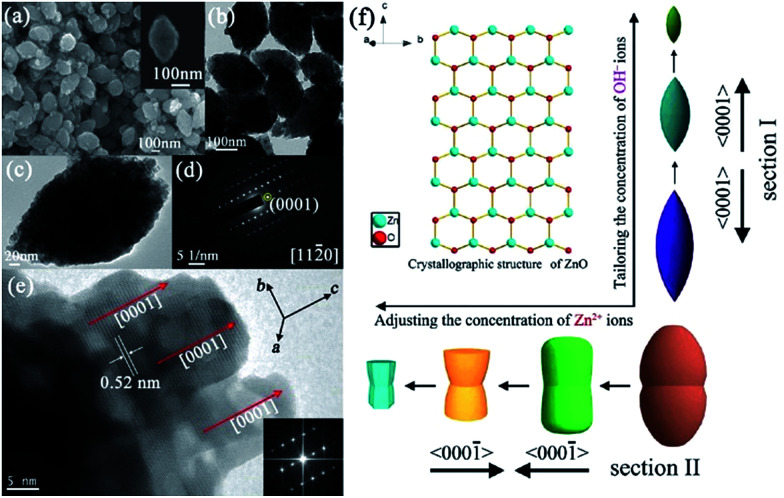
(a) SEM image of the spindle-like ZnO crystals. Inset shows an individual particle. (b) TEM image of the spindle-like ZnO crystals. (c) An individual spindle-like ZnO particle. (d) SAED pattern of the product, as shown in panel (c). (e) HRTEM image of the particle as shown in panel (c); inset shows the corresponding fast Fourier transform (FFT) image. (f) A schematic illustration of the zone-axis preferential growth and reaction pathways of controllable ZnO mesocrystals for different reactant concentrations. Adapted with permission from ref. 80. Copyright 2012 American Chemical Society.

Although the shape-controlled synthesis of ZnO mesocrystals has been achieved by the various abovementioned synthesis methods, the invariable residual organic additives attached to the surfaces of the building blocks resulted in unfortunate problems in their practical applications. Furthermore, the additive-assisted preparation approach not only increased the cost, but also made it more difficult for large-scale synthesis. Hence, it is still a challenge for us to develop new strategies to prepare well-defined ZnO mesocrystals with building blocks as surfactant free as possible. In our previous work, unusual designated tailoring on the zone-axis preferential construction of surfactant-free ZnO mesocrystals with different shapes and sizes was successfully achieved by an additive-free complex-precursor solution method.^80^ The controllable synthesis of ZnO mesocrystals was essentially determined by the characteristic of [Zn(OH)_4_]^2−^ precursors, and an oriented nanoparticle aggregation with tailored sizes and shapes can be generated with different concentrations of reactants at high reaction temperatures. For example, spindle-like ZnO mesocrystals with controllable sizes (along the *c*-axis direction) were prepared by adjusting the concentration of hydroxyl ions, and peanut-like ZnO mesocrystals with tunable sizes (along the *c*-axis direction) and shapes (perpendicular *c*-axis direction) were synthesized by tailoring the concentration of zinc ions (Fig. 9).^80^ The investigation assumes significance in the bottom-up assembly of controllable ordering structures, and it offers a new opportunity to understand the growth mechanism and fundamental significance of zone-axis preferential construction of ZnO mesocrystals. Further, it might provide a green approach to design novel surfactant-free metal oxide mesocrystals with well-defined shapes. Dong and coworkers have developed an ultrafast antisolvent method for the synthesis of spindle-like ZnO mesocrystals.^81^ A deep eutectic solvent, generated by simply mixing and heating urea and choline chloride at 70 °C, can act as the anti-solvent to trigger the ultrafast formation of ZnO mesocrystals. The as-prepared spindle-like ZnO mesocrystals possessed mesoporous and near-single-crystalline characteristic with high specific surface areas, leading to excellent photocatalytic activity toward the photodegradation of methylene blue.

#### 2.1.3 CuO mesocrystals

**Fig. 10:**
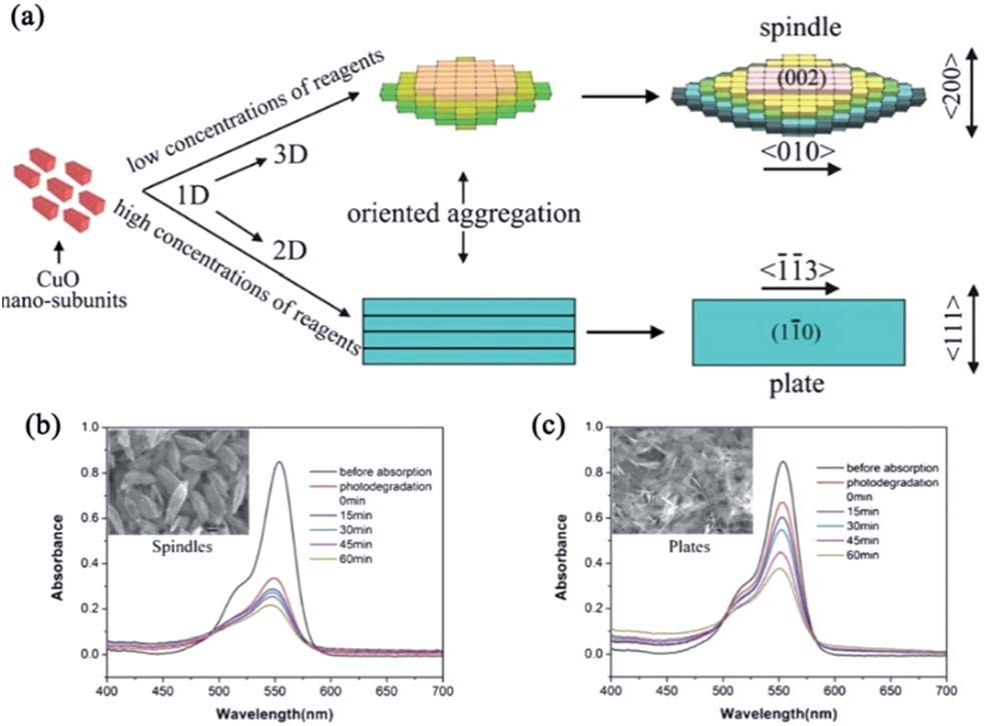
(a) Schematic illustration of the reaction pathway and the ordered-aggregation-driven growth from surfactant-free 1D CuO nanocrystals into dimension-controlled mesostructure (3D mesospindles and 2D mesoplates). (b) and (c) Absorption spectra of the photodegradation of rhodamine B by 3D CuO mesospindles and 2D mesoplates, respectively. Adapted with permission from ref. 86. Copyright 2013 Royal Society of Chemistry.

As a p-type semiconductor with a narrow bandgap varying between 1.2–1.8 eV, CuO has been extensively studied as a photocatalyst or cocatalyst in the past. Various kinds of CuO architectures,^82^ including the ordered aggregation of nano-subunits into CuO mesocrystals has been reported. However, similar to the above TiO_2_ and ZnO mesocrystals, the synthesis of CuO mesocrystals often involves nanocrystal building blocks sharing the same crystallographic orientations with interspersed organic additives. For example, a dandelion-like CuO mesocrystals assembly of rhombic nanoribbons was fabricated by the solvent hydrothermal route in the presence of ethanol.^83^ 3D ellipsoidal CuO mesocrystals composed of a hundred 1D nanoparticles were prepared by a simple solution oxidative route with the assistance of formamide.^84^ 2D CuO mesoplates have been synthesized by using a mild solution in the presence of tetrabutylammonium hydroxide.^85^ Therefore, it is still a challenge to employ additive-free strategies to prepare well-defined CuO mesocrystals with surfactant-free building blocks. In our previous report, based on the synthesis principle of ZnO spindle mesocrystals, we have further developed the green synthesis of ordered-aggregation-driven growth from surfactant-free 1D CuO nano-subunits into dimension-controlled mesostructures (3D mesospindles and 2D mesoplates) by an additive-free complex-precursor solution route.^86^ The formation of CuO mesocrystals was determined by the characteristic of [Cu(OH)_4_]^2−^ precursors, and the oriented aggregation of nanoparticles with well-defined shapes in different dimensions was achieved in different concentrations of reactants at higher reaction temperatures. The 3D “layer-by-layer” growth of mesostructural CuO spindles was successfully prepared in low concentrations of reagents, while the 2D “shoulder-by-shoulder” growth of mesostructural CuO plates was synthesized in high concentrations of reagents. In addition, we further demonstrated that such CuO mesocrystals could serve as a potential photocatalyst for the degradation of rhodamine B under visible-light irradiation in the presence of hydroxide water. The results indicated that 3D mesostructural CuO spindles exhibited higher adsorption and photocatalytic degradation of rhodamine B than that for 2D mesostructural CuO plates (Fig. 10).^86^

#### 2.1.4 Ta_2_O_5_ mesocrystals

**Fig. 11:**
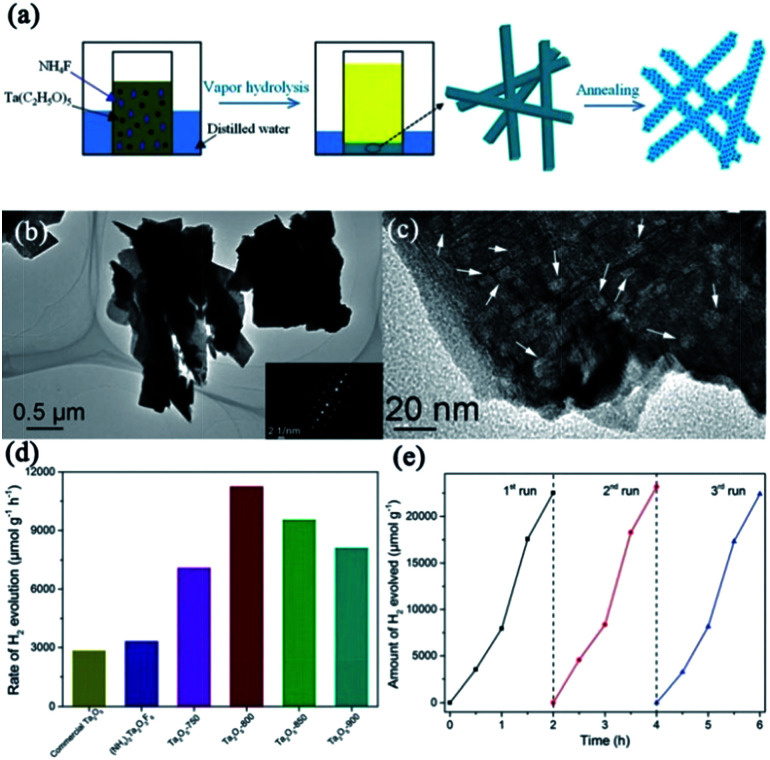
(a) Schematic illustration for the preparation of mesocrystalline Ta_2_O_5_ nanosheets. (b) and (c) TEM and HRTEM images of mesocrystalline Ta_2_O_5_-800 nanosheets (annealed at 800 °C), respectively. (d) Photocatalytic hydrogen evolution rates of commercial Ta_2_O_5_, mesocrystalline (NH_4_)_2_Ta_2_O_3_F_6_ nanorods, and mesocrystalline Ta_2_O_5_ nanosheets. (e) Recyclable photocatalytic performance of mesocrystalline Ta_2_O_5_ nanosheets. Adapted with permission from ref. 88. Copyright 2018 Royal Society of Chemistry.

As a typical wide bandgap semiconductor, Ta_2_O_5_ has a higher CB minimum (CBM) than TiO_2_, which is an obvious advantage for photocatalysis because it potentially provides a strong driving force for water splitting.^87^ Therefore, the construction of superstructured Ta_2_O_5_ mesocrystal nanosheets is highly desirable for exploring efficient and stabilized photocatalysis. In 2018, a study on the synthesis of Ta_2_O_5_ mesocrystals has been reported, in which mesocrystalline Ta_2_O_5_ nanosheets were successfully prepared through the decomposition of mesocrystalline (NH_4_)_2_Ta_2_O_3_F_6_ nanorods by annealing treatment for the first time, as shown in Fig. 11.^88^ The as-synthesized mesocrystalline Ta_2_O_5_ nanosheets exhibited remarkable visible-light absorption, owing to the formation of oxygen vacancy defects in the mesocrystalline nanosheets. When the photocatalytic activity was evaluated, these mesocrystalline Ta_2_O_5_ nanosheets displayed highly photocatalytic hydrogen evolution activity of 11268.24 μmol g^−1^ h^−1^, which was about 3.95 times that of commercial Ta_2_O_5_. This can be attributed to the higher specific surface area and strong oxidizing ability of mesocrystalline Ta_2_O_5_. These mesocrystalline superstructures contributed toward the generation of long lifetime photoinduced carriers and effective conductive pathways for photocatalytic hydrogen production.

### 2.2 Ternary oxide mesocrystals

#### 2.2.1 BiVO_4_ mesocrystals

**Fig. 12:**
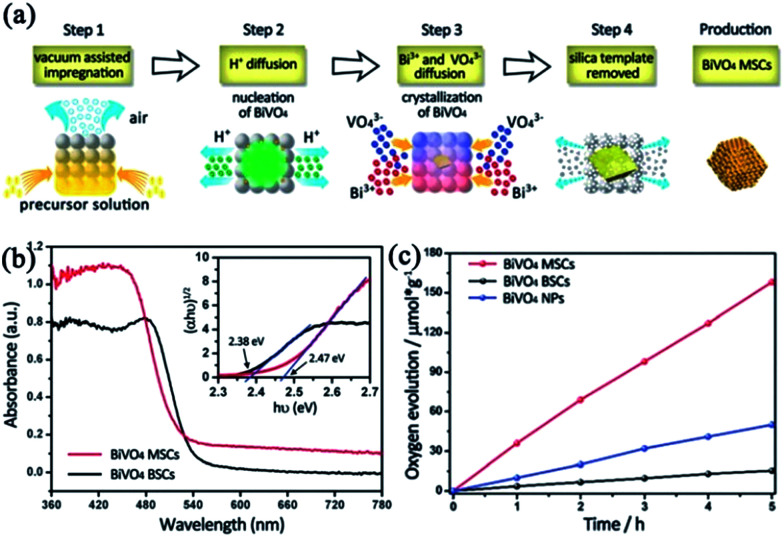
(a) Schematic illustration of the formation mechanism of BiVO_4_ mesoporous single crystals (MSCs). (b) UV-vis diffuse reflectance spectra of BiVO_4_ bulk single crystals (BSCs) (black line) and BiVO_4_ MSCs (red line). The inset shows the plots of (*αhν*)^1/2^*versus* photon energy (*hν*) of the two samples. (c) Photocatalytic oxygen evolution of BiVO_4_ MSCs, BSCs, and nanoparticles. The transient photocurrent and photocatalytic oxygen evolution were conducted using a 300 W Xe lamp (420 nm cut-off filter) as the light source. Adapted with permission from ref. 95. Copyright 2016 Royal Society of Chemistry.

As an ideal semiconductor for photocatalytic oxygen evolution, bismuth vanadate (BiVO_4_) has received much research interest due to its appropriate valence band (VB) edge, narrow bandgap for visible-light absorption, low cost, and good stability.^89,90^ However, its low charge transportation efficiency generally results in a very high electron–hole recombination rate before the electrons and holes reach the interfaces.^91^ Hence, the actual water oxidation efficiency of BiVO_4_ is always much lower than the theoretical value.^92–94^ Therefore, it is imperative to develop suitable methods to enhance the charge transportation efficiency of BiVO_4_. In 2016, BiVO_4_ mesoporous single crystals were successfully prepared, for the first time, by a one-pot hydrothermal method using acidified BiVO_4_ precursor solution pre-impregnated with silica as the template.^95^ The authors proposed a double-diffusion mechanism to illustrate the formation of mesoporous BiVO_4_ single crystals (Fig. 12a). Initially, the diffusion of acid from the silica interior to the bulk solution triggered the nucleation of BiVO_4_ at the interior surface of the silica template. Subsequently, the diffusions of Bi^3+^ and VO_4_^3+^ ions from the bulk solution to the silica template interior resulted in the growth of BiVO_4_ nuclei into single crystals containing the silica template. Finally, mesocrystalline BiVO_4_ mesoporous single crystals were formed based on an oriented attachment and Ostwald ripening mechanism. When compared with BiVO_4_ bulk single crystals, mesoporous BiVO_4_ single crystals exhibited obvious light absorption enhancement in both UV- and visible-light regions, which was attributed to the fact that the inner pores acted as light scattering centers to localize and capture the incident light. Notably, the absorption edge of mesoporous BiVO_4_ single crystals exhibited a distinct blue-shift when compared with that of BiVO_4_ bulk single crystals (Fig. 12b), owing to the contribution of Bi to the VB induced by local structure distortion and quantum size effect. As for mesoporous BiVO_4_ single crystals, the highly crystalline structure was beneficial to the transfer of photogenerated charge carriers in the interior. Further, the mesoporous structure not only promoted the interface transfer of charge carriers, but also reduced the carrier transfer distance in the matrix. As a result, the photocatalytic oxygen evolution rate over mesoporous BiVO_4_ single crystals was improved nearly 10 times than that over bulk single crystals (Fig. 12c).

#### 2.2.2 BaZrO_3_ mesocrystals

**Fig. 13:**
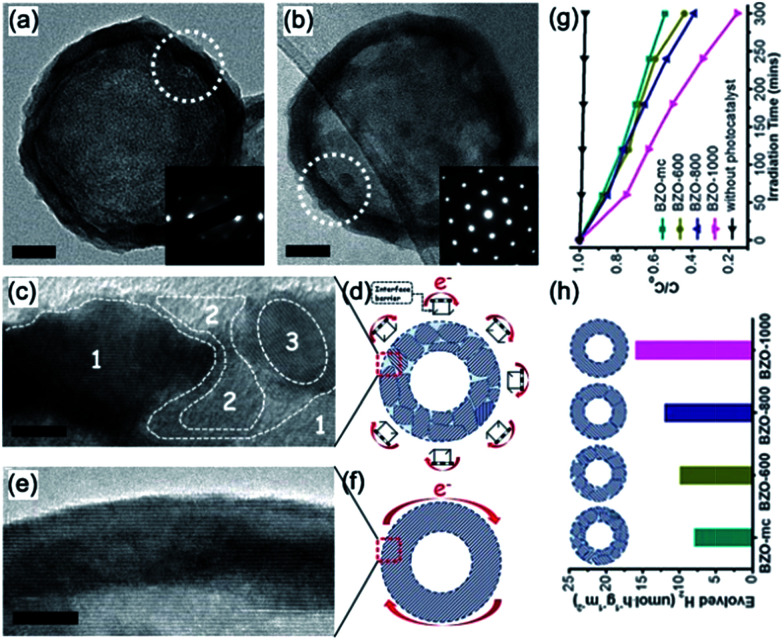
Typical TEM images of individual hollow nanospheres (a) BZO-mc and (b) BZO-1000. Insets (a and b): corresponding SAED patterns of the white dotted cycles; (a and b) scale bars: 20 nm. HRTEM images and corresponding schematic models of the (c and d) BZO-mc and (e and f) BZO-1000 shells. In (d and f), the e^−^ and red arrows represent the photogenerated electrons that were transferred around the outer surface of the hollow nanospheres. Inset (c): area 1 denotes the host lattice and areas 2 and 3 denote the disordered domains. Inset (d): the “hurdle frames” represent the interface barrier among the outer surface grain boundaries. (c and e) Scale bars: 5 nm. (g) Typical photocatalytic activities for hydrogen evolution, and (h) methyl orange degradation curves of BZO-mc, BZO-600, BZO-800, and BZO-1000, respectively. Adapted with permission from ref. 98. Copyright 2014 Royal Society of Chemistry.

As a typical cubic perovskite oxide, BaZrO_3_ is a promising photocatalytic material for water splitting.^96,97^ Generally, the presence of defects among the grain boundaries can serve as trapping and recombination centers between photoinduced electrons and holes, leading to a decrease in the photocatalytic activity.^54,55^ Improving the crystallinity is a possible measure to overcome this drawback. Thus far, investigations on the direct correlation between the crystallinity of BaZrO_3_ mesocrystals (denoted as BZO-mc) and photocatalytic activity is of significance for the construction of highly efficient photocatalysts. However, engineering the crystallinity of semiconductor mesocrystals is rare because the crystal structure of BaZrO_3_ remains stable even at 1000 °C (denoted as BZO-1000). Ye and coworkers have demonstrated the direct evidence of the crystallinity effect of mesocrystals on the photoconversion efficiency by using BaZrO_3_ hollow nanospheres as an ideal photocatalyst model (Fig. 13a–f).^98^ The authors found that the lower recombination rate of the photogenerated charge carriers in the highly crystalline photocatalyst was in favor of enhancing the photocatalytic activities, including high photocatalytic hydrogen production and methyl orange degradation. As shown in Fig. 13g and h, both the hydrogen production rate and degradation rate of methyl orange increased with the crystallinity of BZO-mc. This work presents a better understanding of the actual crystallinity effect on the photocatalytic performance of mesocrystal photocatalysts.

#### 2.2.3 NaTaO_3_ mesocrystals

**Fig. 14:**
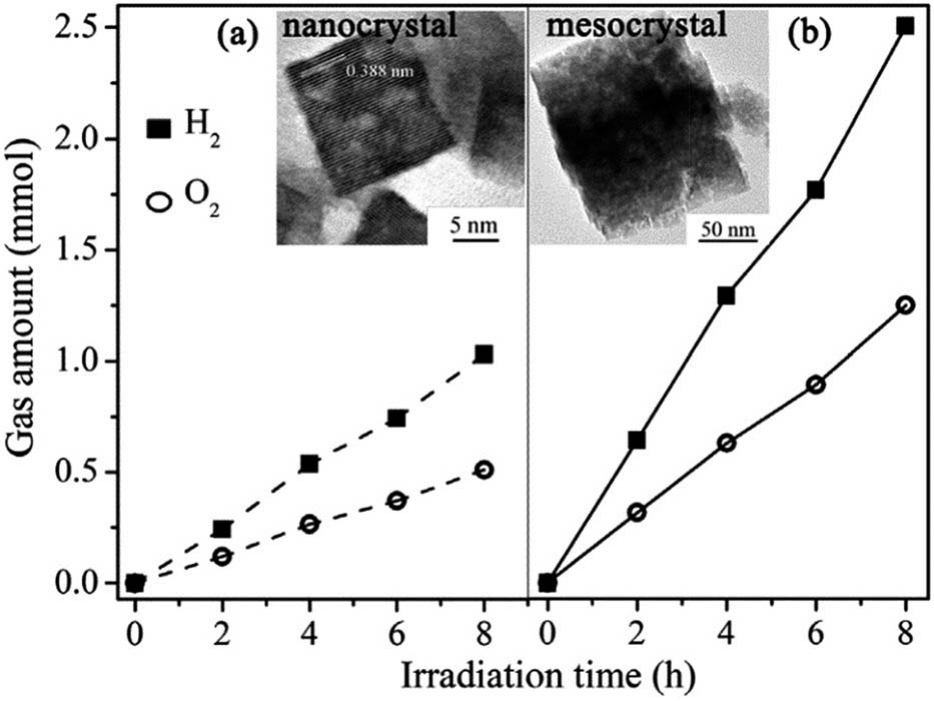
Photocatalytic water splitting for hydrogen and oxygen generation. (a) Nanocrystals (dashed line) and (b) NaTaO_3_ mesocrystals (solid line). Adapted with permission from ref. 101. Copyright 2013 Elsevier.

Perovskite sodium tantalate (NaTaO_3_) is the most efficient water-splitting catalyst reported with a bandgap up to 4.01 eV.^99,100^ To optimize its photocatalytic performance, several strategies were employed to alter the band structure, phase structure, particle morphology, and size of NaTaO_3_. In particular, mesocrystalline NaTaO_3_ nanoparticles were confirmed for effectively improving the photocatalytic activity.^101^ Cubic NaTaO_3_ mesocrystals with sub-100 nm sizes and high crystallinity were synthesized in a surfactant-free environment by alkoxide-hydrolyzation-based rapid reaction. The self-assembly of primary nanoparticles was controlled by adjusting the concentration of reagents, and larger mesocrystals with pores were prepared under higher concentrations. The as-produced mesocrystalline NaTaO_3_ exhibited a surface step structure and high surface area with abundant active sites for the improvement of charge separation and migration, which resulted in a photocatalytic hydrogen evolution rate as high as 3.106 mmol h^−1^ g^−1^, as shown in Fig. 14.^101^

#### 2.2.4 SrTiO_3_ mesocrystals

**Fig. 15:**
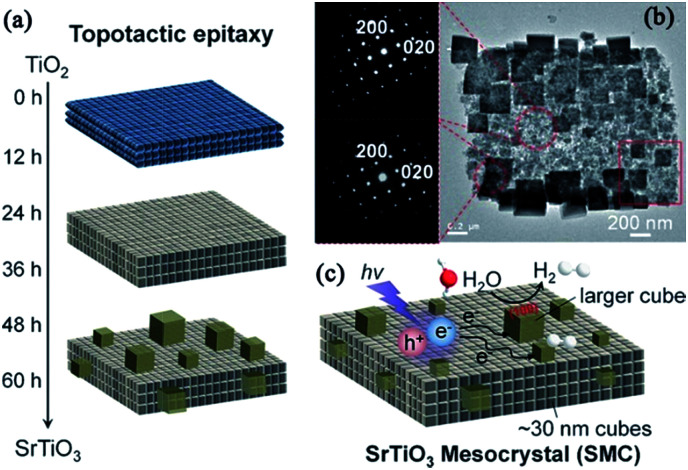
(a) Schematic illustration of the topotactic epitaxy of SrTiO_3_ mesocrystals from TiO_2_ mesocrystals. (b) TEM image of SrTiO_3_ mesocrystals (reaction time: 48 h) with SAED from near the center and at the edge (red circle). (c) Anisotropic electron transport from the inside to the outside of SrTiO_3_ mesocrystals comprising aligned nanocubes with dominant (100) facets. The symbols e^−^ and h^+^ indicate photogenerated electrons and holes, respectively. Adapted with permission from ref. 105. Copyright 2017 Wiley-VCH Verlag GmbH & Co.

As a typical cubic-perovskite-type multimetallic oxide with a bandgap of 3.2 eV, strontium titanate (SrTiO_3_) is a promising photocatalyst to split water into hydrogen and oxygen and to degrade organic pollutants, because of its extraordinary physical and chemical properties such as excellent thermal stability, photocorrosion resistibility, and good structural stability.^102,103^ Consequently, the successful synthesis of mesocrystalline SrTiO_3_ structures with high crystallinity would potentially improve its photocatalytic performance. Based on the previous literature, it can be found that the hydrothermal topotactic epitaxy of titanate was an effective strategy for the controllable synthesis of SrTiO_3_ mesocrystals.^104,105^ For instance, Majima and coworkers have prepared a novel perovskite SrTiO_3_ mesocrystal superstructure with well-defined orientation of assembled cubic nanocrystals induced by topotactic epitaxy from TiO_2_ mesocrystals through a facile hydrothermal treatment.^105^ In this case, starting from sheet-like TiO_2_ mesocrystals, which consisted of assembled anatase TiO_2_ nanocrystals with dominant (001) facets, Sr^2+^ ions from Sr(OH)_2_ reacted with TiO_2_ in an alkaline solution to generate an epitaxial overlayer of SrTiO_3_ grains at the interface *via* the dissolution–precipitation process. During the nucleation and growth (12–24 h) stages, cubic SrTiO_3_ nanocrystals with highly ordered orientation were formed, followed by an increase in crystallinity. Upon prolonging the hydrothermal time to 36 h, larger cubic crystals were generated onto the external surfaces of SrTiO_3_ mesocrystals. A schematic shape evolution of the SrTiO_3_ mesocrystals is shown in Fig. 15a. As shown in Fig. 15b, the typical TEM image indicates that the external surfaces of SrTiO_3_ mesocrystals are covered with larger nanocubes (200 nm) rather than small nanocubes (30 nm) assembled inside the mesocrystals. The corresponding SAED patterns obtained near the center and edge (red circles) exhibit single-crystal-like spots, suggesting that SrTiO_3_ mesocrystals are made of crystallographically aligned nanocrystals. It should be noted that the orientation of crystals is maintained during the epitaxial structural conversion and subsequent crystal growth, which is obviously attributed to particle-to-particle interactions in the mesocrystal. According to the structural features of the above SrTiO_3_ mesocrystals, the authors have proposed the photocatalytic mechanism and charge transfer process (Fig. 15c) in which the well-ordered architecture promoted the spatial charge separation throughout the long-distance electron transfer along the highly ordered adjacent nanocubes. Therefore, the photogenerated charge carriers were transported to the larger external nanocubes with active (100) facets, where hydrogen ions were reduced to generate hydrogen gas under UV-light irradiation. The morphology of the titanate precursors is significant in the controllable synthesis of SrTiO_3_ mesocrystals. Kalyani and coworkers have prepared 1D rod-like SrTiO_3_ mesocrystals through the hydrothermal crystallization from the suspensions of single-crystal anatase and hydrogen titanate nanowires, which further revealed the mechanism driving the mutual crystallographic alignment of nanocrystals in mesocrystals. In this synthesis, the lattice mismatch and defective state of the precursor surface might determine the degree of mutual crystallographic alignments of the subunits, namely, the larger lattice mismatch would result in a lower level of ordering.^106^

In addition, single-crystal-like mesoporous SrTiO_3_ sub-micrometer spheres with large surface area and high crystallization were successfully produced by a facile hydrothermal approach in the presence of tetrabutyl titanate/strontium nitrate/potassium hydroxide/polyvinyl alcohol (PVA) system.^107^ The oriented aggregation of nanoparticles was proposed to be the dominant formation mechanism, which was accompanied by the ripening process. Typically, the pore density of the as-prepared SrTiO_3_ spheres obviously increased as the PVA concentration increased, and the average pore size ranged from 4.5 to 16.1 nm. The photocatalytic degradation of rhodamine B with the as-produced mesocrystalline SrTiO_3_ spheres was a function of PVA concentration and reaction time. The highest photocatalytic activity has been achieved in mesocrystalline SrTiO_3_ synthesized at 200 °C for 6 h with a higher PVA concentration.

#### 2.2.5 In_2_O_3−*x*_(OH)_*y*_ mesocrystals

**Fig. 16:**
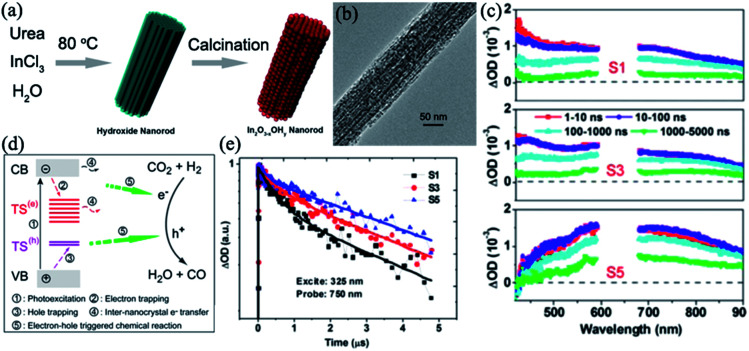
(a) Schematic illustration of the synthesis of rod-like In_2_O_3−*x*_(OH)_*y*_ mesocrystals. (b) Typical TEM image of a mesocrystalline In_2_O_3−*x*_(OH)_*y*_ rod. (c) Time-resolved absorption spectra (nanosecond to microsecond range) observed after 325 nm laser pulse excitation of different In_2_O_3−*x*_(OH)_*y*_ samples in N_2_ gas. (d) Schematic illustrations of the photoexcited electron–hole dynamics and migration of a photogenerated electron between neighboring nanocrystals. Surface trapping states and interparticle charge transfer are in favor of the spatial separation of electron–hole pairs, which promotes the photo-redox reaction. (e) Normalized transient absorption traces observed at 750 nm for S1 (synthesis time = 2 h), S3 (synthesis time = 3 h), and S5 (synthesis time = 5 h). Adapted with permission from ref. 108. Copyright 2016 American Chemical Society.

Although it has been demonstrated that mesocrystals with long lifetimes of photoexcited charge carriers can exhibit enhanced activity in liquid-phase photocatalytic dye degradation and water splitting, the photocatalytic performances of mesocrystalline superstructures toward gas-phase chemical reactions have not yet been investigated. He and coworkers presented the spatial separation of charge carriers in mesocrystalline In_2_O_3−*x*_(OH)_*y*_ superstructures for enhanced gas-phase photocatalytic activity in 2016,^108^ which were synthesized through a two-step process, including the synthesis of In(OH)_3_ nanorods using InCl_3_ and urea as the precursors without initially using any surfactant, followed by the transformation into bixbyite-structured indium oxide (Fig. 16a).

A typical nanorod exhibiting a nanoporous superstructure is shown in Fig. 16b. It has been demonstrated that interparticle charge transfer generated within the nanocrystal superstructure and the lifetime of photoinduced carriers was prolonged in In_2_O_3−*x*_(OH)_*y*_ mesocrystals, which was in favor of the increase in the conversion rate of the gas-phase, light-assisted reverse water–gas shift reaction. Under solar-light illumination, photogenerated electrons from the VB would be excited into the CB of the semiconductor, leading to the formation of photogenerated holes in the VB. The photogenerated holes migrated into the surface hydroxide trap states, while the photogenerated electrons located in the CB might be captured in the oxygen vacancies. Notably, the mesocrystalline In_2_O_3−*x*_(OH)_*y*_ nanorods were made up of close-contact nanocrystals, which would cause the spatial separation of the photoexcited carriers between the neighboring subunits, and the migration of holes between the neighboring nanocrystals has a lower probability than electron movement (Fig. 16c–e).

In addition, it is interesting to note the nanorod length dependence on the hydrogenation rate of carbon dioxide to carbon monoxide.

#### 2.2.6 Nb_3_O_7_(OH) mesocrystals

**Fig. 17:**
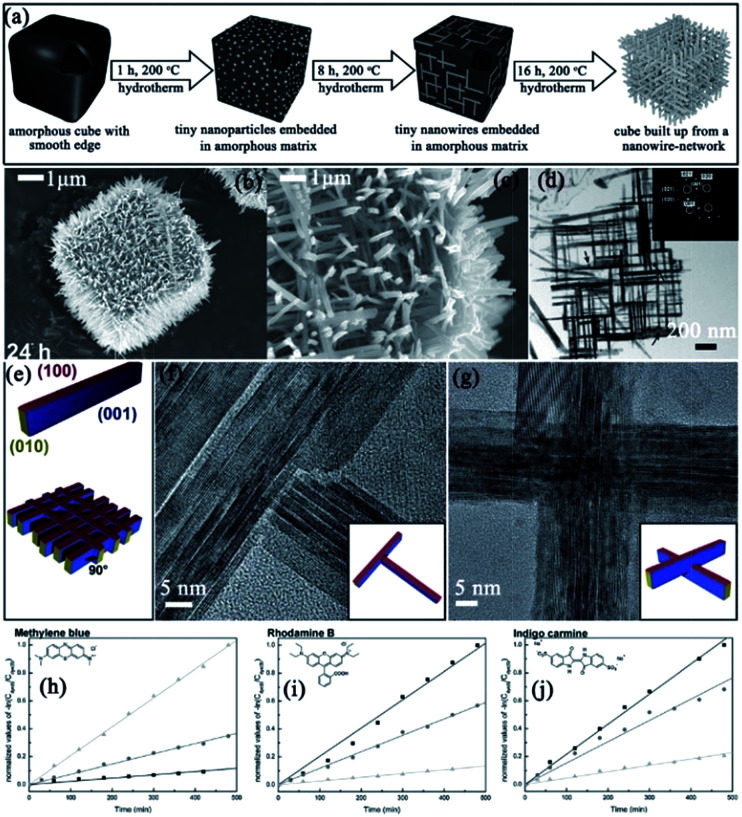
(a) Schematic illustration of the hydrothermal growth of Nb_3_O_7_(OH) mesocrystals. (b) and (c) Low- and high-magnification SEM images, respectively. (d) TEM image of a fragment of one cube wall; the inset shows the corresponding SAED pattern. (e) Schematic drawing illustrating the crystal shape of the nanowires and crystallographic arrangement of the nanowires in the network. (f) HRTEM image of a T-shaped nanowire junction and schematic illustration showing the arrangement of the nanowires at the junction (inset). (g) HRTEM image of a nanowire crossing and schematic drawing of the junction (inset). (h)–(j) Measurement of the photocatalytic degradation of three different dyes at three different pH values (pH 2 (■), pH 6 (●), and pH 10 (▲)). The kinetic rate constant can be determined from the curve obtained by plotting −ln(*C*_dye_/*C*_0_) *versus* the irradiation time *t*. The corresponding curves are shown in (h) for methylene blue, in (i) for rhodamine B, and in (j) for indigo carmine. Adapted with permission from ref. 109. Copyright 2014 Royal Society of Chemistry.

Niobium oxide, such as Nb_3_O_7_(OH) mesocrystals, is a promising material for photochemical and photophysical devices. Betzler and coworkers have developed a template-free hydrothermal synthesis approach for preparing 3D hierarchical Nb_3_O_7_(OH) superstructures with perpendicularly aligned nanowire building blocks (Fig. 17).^109^ The as-synthesized products were synthesized by the hydrothermal treatment of an aqueous Nb(iv) chloride solution at temperatures between 150 and 200 °C, and the morphologies can be tailored by adjusting the synthesis parameters including the pH value, temperature, and reaction time. Based on the time-dependent shape-evolution process, it can be found that mesocrystalline Nb_3_O_7_(OH) nanowire networks resulted from the transformation of amorphous hollow cubes with smooth surfaces under hydrothermal conditions. The obtained cubic superstructures with a bandgap of 3.2 eV possessed high photocatalytic degradation efficiency toward different organic dyes because of the periodic nanowire networks, large surface area, and high crystallinity.^109^

### 2.3 Quaternary mesocrystals

**Fig. 18:**
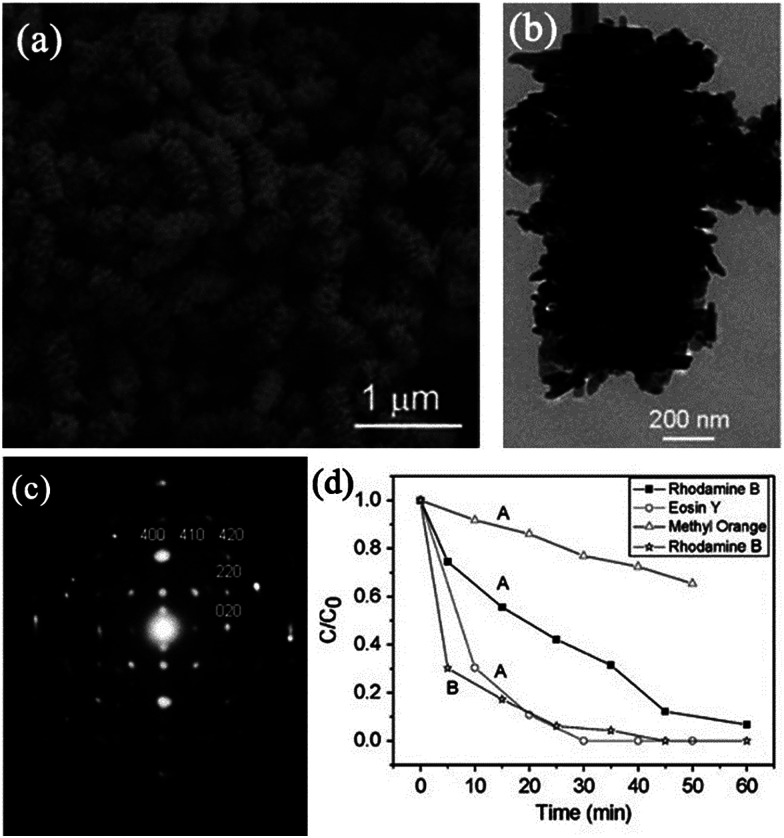
(a) SEM image of caterpillar-like AgIn(WO_4_)_2_ mesocrystals. (b) TEM image of an individual caterpillar-like particle. (c) Corresponding SAED pattern. (d) Photocatalytic degradation of different organic dyes under 300 W Xe lamp irradiation with AgIn(WO_4_)_2_ mesocrystals. Adapted with permission from ref. 110. Copyright 2010 Royal Society of Chemistry.

Thus far, quaternary metal oxide mesocrystals have been rarely reported. AgIn(WO_4_)_2_ is a typical example. Yu and coworkers have reported a facile and mild microwave-assisted route for synthesizing caterpillar-like AgIn(WO_4_)_2_ mesocrystals with tuned shapes in the presence of AgNO_3_, In(NO_3_)_3_, and Na_2_WO_4_.^110^ In this synthesis, microwaves might improve the nucleation and growth of AgIn(WO_4_)_2_ in the Ag/In/W/O system. Initially, small amorphous nanoparticles were generated and randomly assembled in an aggregative manner. Subsequently, these connected nanoparticles evolved into tiny olive-like core structures by the oriented-attachment process. Finally, outgrowths occurred on the surface of the core and produced caterpillar-like architectures under the oriented-attachment process accompanied by Ostwald ripening. It should be noted that the pH value played an important role in the phase formation and morphology evolution. These caterpillar-like AgIn(WO_4_)_2_ mesocrystals displayed selective photocatalytic properties for degrading organic dyes under UV- and visible-light irradiation. The rate constants for the degradation of eosin Y, rhodamine B, and methyl orange were 0.111, 0.044, and 0.0084 min^−1^, respectively, which clearly demonstrated that the photodegradation process of eosin Y was much faster than those of rhodamine B and methyl orange (Fig. 18).^110^ Moreover, the photoluminescence spectra of AgIn(WO_4_)_2_ mesocrystals with different morphologies have been investigated by the above authors. It can be found that all the products displayed white emission in the visible region when excited by visible light with a wavelength of 460 nm because of the surface nanostructures of the outgrowths.^111^

Based on the above overview, the synthesis strategies of diversified photocatalyst mesocrystals are an exciting direction to fabricate compounds with high activity. Further, it provides an opportunity to investigate structure-related photocatalytic performance relationship. However, it should be noted that a series of hybrid mesocrystal-based heterogeneous photocatalysts with well-controlled compositions, shapes, and sizes have been demonstrated in the field of photocatalysis along with rapid progresses in nanomaterials science and nanotechnology.

## 3 Functionally modified mesocrystal photocatalysts

Effectively understanding the correlations between the modified interfacial/electronic structures and improved photocatalytic performances is crucial for developing novel mesocrystal-based photocatalysts. Generally, the modifications of mesocrystal photocatalysts can be divided into two strategies: hybridization and doping. Hybridization is a general strategy for inducing unexpected physicochemical characteristics to improve the potential application of a single material, which is attributed to the synergistic effect between the active component and support.^112^ Therefore, mesocrystals acting as host components would offer a good chance to tune the interfacial property of hybrid mesocrystal-based micro/nanostructures for improving practical applications. Furthermore, an alternative strategy for improving the physicochemical properties is to dope heteroatoms into the mesocrystal to modify its electronic structure. However, a systematic review of mesocrystal-based architectures has not been reported so far. In this section, we will firstly summarize the significant advances in the development of different types of hybrid mesocrystal-based photocatalysts, such as hybrid semiconductor–mesocrystals, hybrid mesocrystal–metal nanostructures, and hybrid mesocrystal–carbon nanostructures. Next, doped mesocrystal photocatalysts will be introduced based on some typical examples.

### 3.1 Hybridization

#### 3.1.1 Semiconductor–mesocrystals

**Fig. 19:**
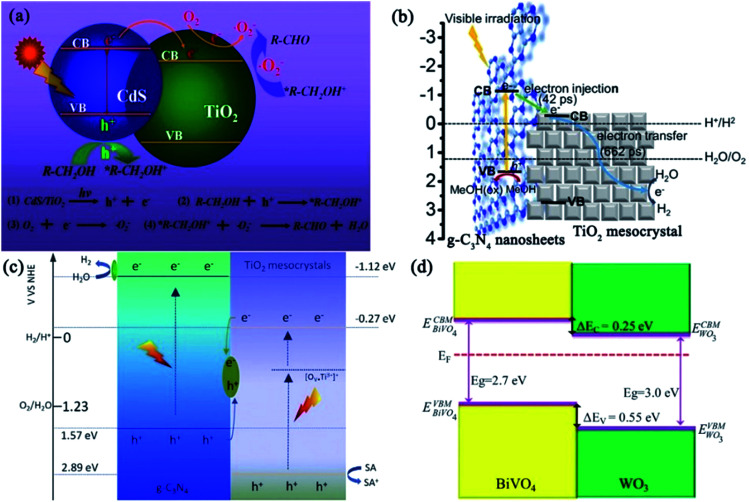
(a) Schematic illustration of the CdS photosensitizing effect, photogenerated electron transfer from CdS to TiO_2_ mesocrystal *via* the heterojunction, and mechanism of photocatalytic selective oxidation of alcohols into aldehydes. Adapted with permission from ref. 113. Copyright 2016 Elsevier. (b) Representative scheme of photogenerated electron injection and movement in g-C_3_N_4_ nanosheet (31 wt%)/TiO_2_ mesocrystals under visible-light irradiation. Adapted with permission from ref. 114. Copyright 2017 American Chemical Society. (c) Possible visible-light photocatalytic mechanism of Ti^3+^-doped mesocrystalline TiO_2_/g-C_3_N_4_ composites for hydrogen production. Adapted with permission from ref. 115. Copyright 2018 Elsevier. (d) Band alignment of BiVO_4_/WO_3_ heterojunction. *E*_VBM_ is the VBMs, *E*_CBM_ is the CB minima, and Δ*E*_V_ and Δ*E*_c_ are the VB and CB offsets, respectively. Adapted with permission from ref. 116. Copyright 2017 Nature Publishing Group.

The controllable synthesis of heterogeneous semiconductor–mesocrystal composites is an exciting direction to pursue highly active photoelectric beacons, and it also provides a good opportunity to investigate the structure–performance relationship. Recently, considerable efforts have been employed to construct high-activity hybrid semiconductor–mesocrystals with various heterogeneous interfaces for photocatalysis.^113–117^ In this section, we will mainly summarize the enhanced photocatalytic mechanism of different mesocrystalline TiO_2_/semiconductors according to previous reports because other composites have not been reported so far. The combination of TiO_2_ mesocrystals with photosensitizers can enhance the light-harvesting ability of TiO_2_ under solar-light irradiation, achieving visible photocatalysis. Bian and coworkers have presented, for the first time, novel TiO_2_ mesocrystals with exposed (001) facets synthesized from a simple solvothermal alcoholysis process, followed by modification with CdS quantum dots (narrow energy bandgap: ∼2.4 eV) through a facile ion-exchange treatment.^113^ The integration of mesoporous anatase TiO_2_ mesocrystals with exposed (001) facets and CdS photosensitizing effects led to high photocatalytic performance for the selective oxidation of alcohols to aldehydes under visible-light irradiation. The good photocatalytic efficiency was attributed to CdS quantum dots with improved photosensitizing effect and CdS/TiO_2_ heterojunctions; meanwhile, the mesoporous structure with high surface area and exposed (001) facets with high surface energy, as well as the large amount of oxygen vacancies, enhanced the light-harvesting capacity, photoinduced charge carriers separation capability, reactant molecule adsorption, and activation characteristics. In this system, it has been confirmed that the enhanced number of CdS/TiO_2_ heterojunctions can facilitate the transfer of photoinduced electrons from CdS to TiO_2_ and improve the separation of photoelectrons from holes (Fig. 19a). Moreover, the (001) facets exhibited higher surface energy, which favored the photoactivation of reactant molecules when compared with those for (101) facets. Furthermore, the (001) facets also exhibited strong interactions with CdS nanoparticles, which might optimize the photosensitizing effect of CdS and further accelerate the photoelectron transfer from CdS to TiO_2_*via* heterojunctions. In addition, the (001) facets possessed more oxygen vacancies than the (101) facets, which would trap photogenerated electrons, and therefore, limit the recombination with holes.

The photocatalytic activity of graphitic carbon nitride (g-C_3_N_4_) was inhibited to lower efficiencies because of the fast recombinations of the photogenerated charge carriers. Majima and coworkers have synthesized g-C_3_N_4_ nanosheets/TiO_2_ mesocrystals metal-free composites to improve the charge separation. The photocatalytic hydrogen evolution experiments demonstrated that coupling g-C_3_N_4_ nanosheets (31 wt%) with TiO_2_ mesocrystals exhibited the highest photocatalytic activity, which was 20 times larger than that of pristine g-C_3_N_4_ without any noble metal cocatalyst under visible-light irradiation (*λ* > 420 nm) and 7 times higher than that of g-C_3_N_4_/P25, suggesting the significance of the strong interface interactions between 2D g-C_3_N_4_ nanosheets and plate-like TiO_2_ mesocrystals. Significantly, femtosecond time-resolved diffuse reflectance (fs-TDR) spectra revealed that the migration of photogenerated charge carriers was promoted and their lifetimes were prolonged because of the tight interface between the g-C_3_N_4_ nanosheets and plate-like TiO_2_ mesocrystals. Consequently, the fs-TDR spectra indicated that TiO_2_ mesocrystals acted as an electron transfer channel to promote charge separation (Fig. 19b).^114^ Similarly, direct Z-scheme Ti^3+^ self-doped TiO_2_ mesocrystals/g-C_3_N_4_ composites were synthesized by a simple solvothermal method, which exhibited visible-light absorption and photocatalytic activity for hydrogen production. This direct Z-scheme photocatalytic mechanism is confirmed as follows (Fig. 19c).^115^ Under visible-light irradiation, the Ti^3+^-doped TiO_2_ mesocrystals and g-C_3_N_4_ nanosheets easily excited the photogenerated carriers, and the photogenerated electrons from the VB of g-C_3_N_4_ nanosheets had strong reduction potential when compared with the CB electrons of TiO_2_. They would react with water for hydrogen evolution, and the VB holes in Ti^3+^-doped TiO_2_ possessed strong oxidizing potential and could oxidize the sacrificial agents, but the VB holes of g-C_3_N_4_ could not react with the sacrificial agents due to a lower oxidizing potential. In addition, the CB electrons of Ti^3+^-doped TiO_2_ mesocrystals would react with the generated VB holes of g-C_3_N_4_ owing to the short charge-transfer distance, leading to an improvement in charge separation. In addition, Van and coworkers have demonstrated a self-assembled nanocomposite photoanode composed of an epitaxial BiVO_4_ matrix embedded with WO_3_ mesocrystals for visible-light-driven photoelectrochemical applications. In this system, the orientation of the crystal facets and interfaces offered a superior template to investigate the intimate contact between the two constituent phases, which revealed that the interfacial coupling of the mesocrystal and matrix enhanced the separation and migration of photogenerated carriers (Fig. 19d), leading to a largely improved photoelectrochemical activity when compared with their counterparts.^116^

**Fig. 20:**
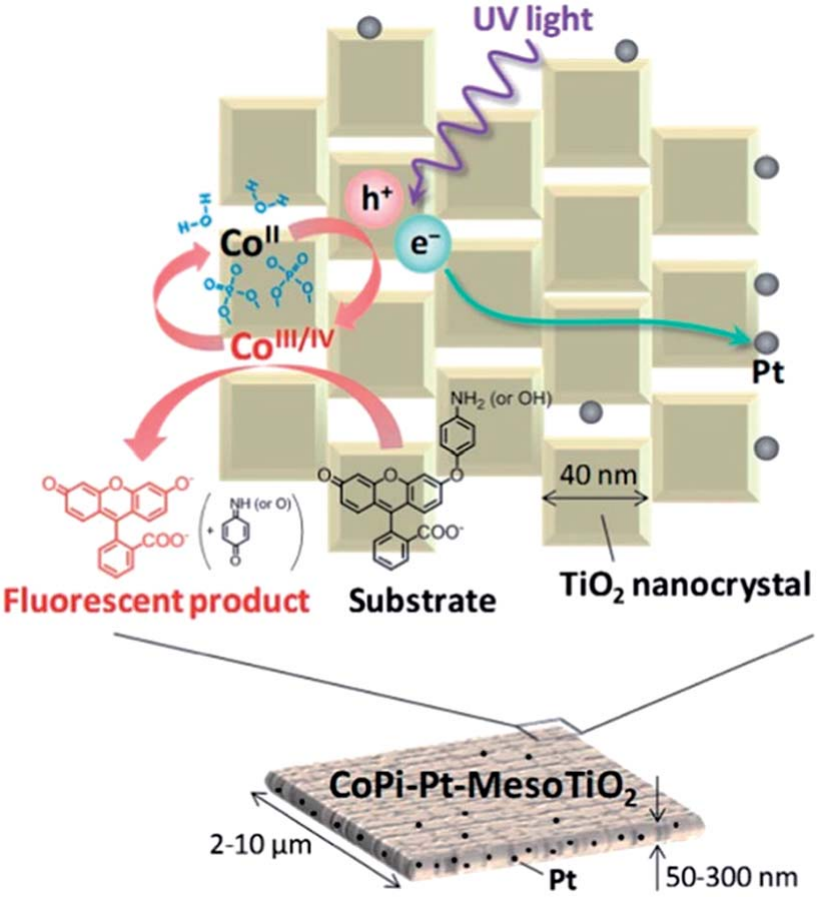
Schematic illustration of the photogenerated charge transfer on the surface of CoPi/Pt/TiO_2_ mesocrystal. Adapted with permission from ref. 117. Copyright 2014 Royal Society of Chemistry.

As an efficient oxygen-evolution cocatalyst, cobalt phosphate (CoPi) has been decorated onto TiO_2_ mesocrystals for enhancing the photocatalytic water-splitting ability. For a CoPi/TiO_2_ mesocrystal/Pt photocatalytic hydrogen evolution system, active Co III/IV species in CoPi, as well as holes in the VB of TiO_2_, can oxidize the probe dye of 3′-*p*-aminophenyl fluorescein and 3′-*p*-hydroxyphenyl fluorescein to produce fluorescein as the fluorescent product through O-dearylation reaction. During this reaction, the photogenerated holes in the VB of TiO_2_ would transfer to the CoPi cocatalysts and get deposited onto the surface of the (001) facets upon UV-light irradiation. The photoexcited electrons in the CB of TiO_2_ preferentially transferred to the lamellar (101) facet to deposit Pt, finally limiting the recombination of charge carriers (Fig. 20).^117^ Moreover, Majima's group has demonstrated that polyhedral TiO_2_ mesocrystals packed with an exfoliated MoS_2_ shell can exhibit promising reactive efficiency and good stability in photocatalytic hydrogen evolution, which is attributable to the anisotropic electron flow to achieve enhanced photocatalytic performance.^118^

#### 3.1.2 Metal–mesocrystals

Mesocrystals with special surface structures can effectively stabilize the as-deposited metal nanoparticles, resulting in the formation of hybridization composites with good stability and improved charge separation. However, the controllable synthesis of hybrid metal–mesocrystal composites is still in its infancy. Consequently, it is necessary to summarize the advances in hybrid metal–mesocrystal composites.

**Fig. 21:**
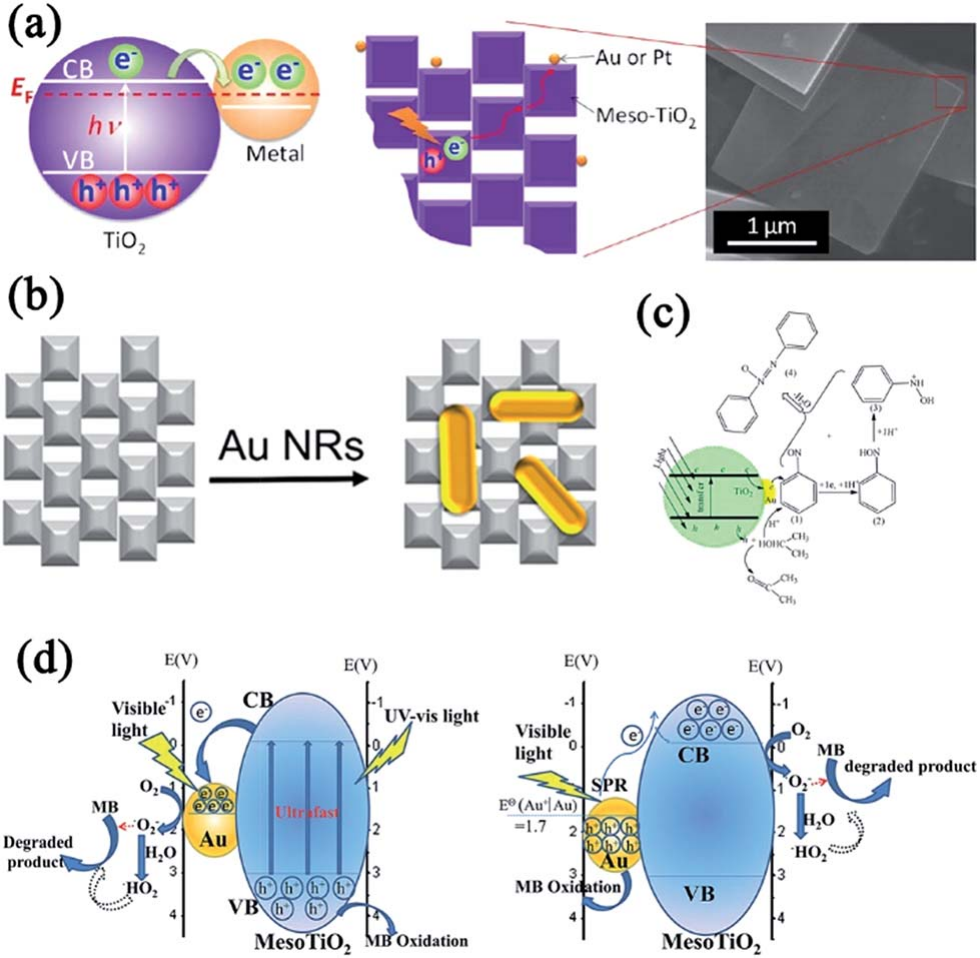
(a) Schematic illustration of electron transfer from TiO_2_ to noble metal (Au, Pt) nanoparticles upon irradiation of UV light, and electron transfer on Au/TiO_2_ mesocrystal or Pt/TiO_2_ mesocrystal. Adapted with permission from ref. 119. Copyright 2012 American Chemical Society. (b) Preparation of rod-like Au/TiO_2_ mesocrystals. Adapted with permission from ref. 121. Copyright 2017 Elsevier. (c) Proposed mechanism for the photocatalytic reduction of nitrosobenzene to azoxybenzene by Au/TiO_2_ mesocrystals. Adapted with permission from ref. 122. Copyright 2016 IOP Publishing. (d) Proposed mechanism for the photocatalytic activity of Au/TiO_2_ mesocrystals under UV-vis-light excitation (left) and visible-light excitation (right). Adapted with permission from ref. 123. Copyright 2016 Royal Society of Chemistry.

It is well known that the Fermi level and electron-accepting states of noble metals are generally located at an energy level below the CB of TiO_2_ semiconductors.^1^ Accordingly, with regard to a noble metal–TiO_2_ system, the photogenerated electrons in the CB of TiO_2_ will effectively migrate to the deposited metal nanoparticles under UV-light irradiation, while the photoexcited holes stay in the VB of TiO_2_, finally resulting in the achievement of charge carrier separation. Moreover, the photoinduced electrons can transfer to TiO_2_ under visible-light irradiation due to the localized surface plasmon resonance.^120^ Notably, TiO_2_ mesocrystals provide good support for depositing noble metals to enhance the photocatalytic activity, because the highly ordered superstructure with high surface area can avoid the numerous interfacial defects, facilitate charge separation as well as transfer, and provide abundant reaction sites for photocatalytic reactions. Bian and coworkers have developed a facile photodeposition strategy to synthesize Au- or Pt-nanoparticle-loaded TiO_2_ mesocrystals, and the transport and reaction dynamics of the photogenerated charge carriers in individual composite materials are investigated.^119^ Based on the single-molecule fluorescence spectroscopy measurements on a single composite particle, it has been found that most of the photoexcited electrons could transfer from the dominant (001) facet to the edge of TiO_2_ mesocrystals in micrometer distances, and the photoreduction reactions mainly occurred at the lateral surfaces containing (101) facets. Therefore, this anisotropic electron flow in the superstructure considerably limited the electron recombinations with holes, leading to improved photocatalytic oxidation activity (Fig. 21a).^119^ More interestingly, TiO_2_ mesocrystals composited with Au nanorods can be used for highly efficient visible-NIR-photocatalytic hydrogen production (Fig. 21b).^121^ Yan and coworkers have deposited Au nanoparticles selectively anchored on the (101) facet of polyhedral TiO_2_ mesocrystals, which exhibited highly selective photocatalytic reduction of nitroarenes because of the plasmonic effect of Au nanoparticles, unique superstructure of TiO_2_ mesocrystals, and highly strong interaction between Au and TiO_2_ through the close Schottky heterointerface (Fig. 21c).^122^ In addition, the detailed electron–hole separation dynamics for visible-light- and UV-vis-induced catalytic mechanisms of Au/TiO_2_ mesocrystals were discussed in detail by Wang and coworkers (Fig. 21d).^123^ Similarly, Ag/hematite mesocrystal composites can exhibit high photo-Fenton activity in the oxidation of rhodamine B, methyl orange, and glyphosate under visible-light irradiation.^124^ Apart from the abovementioned synthesis strategy, wet chemical impregnation method and ion exchange–reduction approach might be useful for the synthesis of metal–mesocrystal composites, but it has not been extensively studied so far.

#### 3.1.3 Carbon–mesocrystals

Carbon nanomaterials, including carbon dots (CDs), graphene, and graphene oxide (GO), have been investigated as excellent supports for photocatalysis because of their outstanding thermal and chemical stabilities, high conductivities, and high specific surface areas. As for carbon–mesocrystals composites, the carbon supports/or active ingredients would serve as good electron acceptors that can promote the charge separation and migration in mesocrystal semiconductors. As a result, improved activities can be achieved for hybrid carbon–mesocrystals composites when compared with pristine mesocrystal semiconductors. In this subsection, we will briefly summarize the different types of hybrid carbon–mesocrystal photocatalysts.

**Fig. 22:**
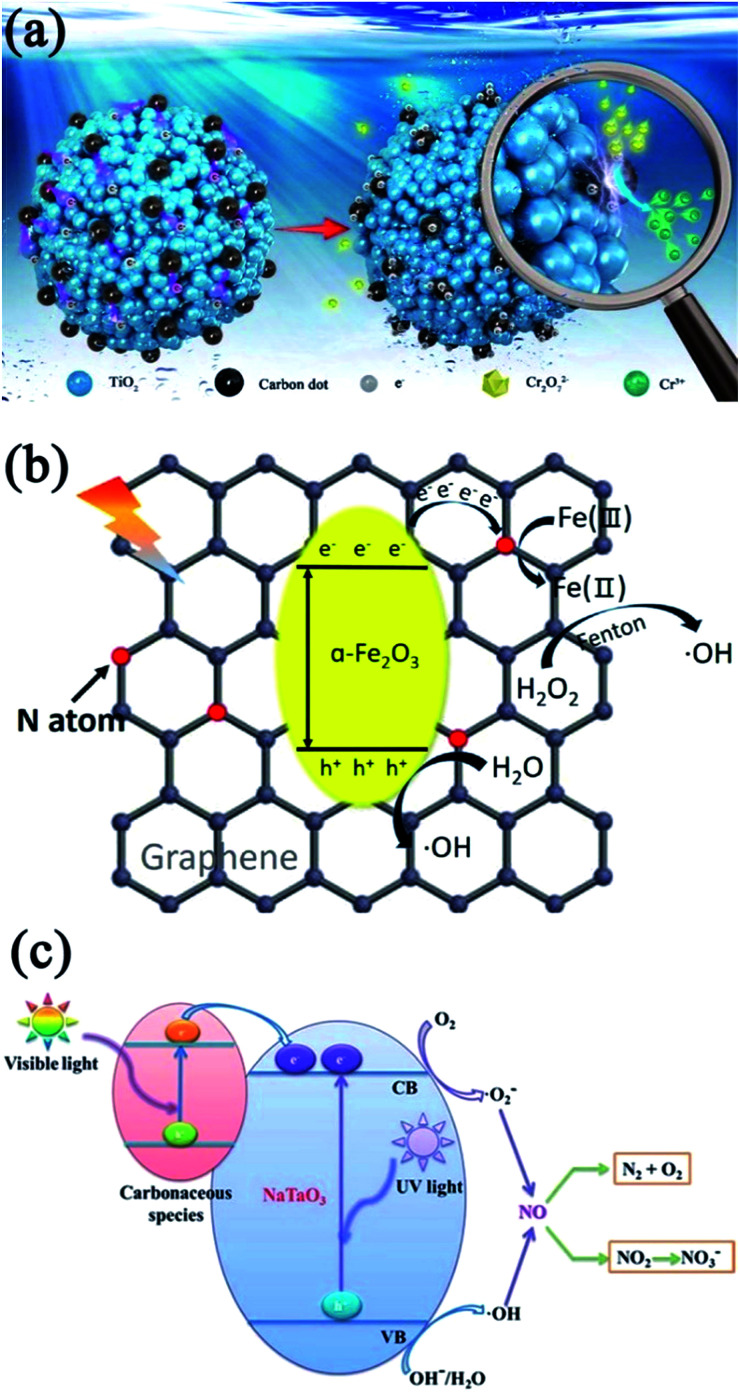
(a) Proposed adsorption–photoreduction desorption mechanisms of photocatalytic reduction of Cr(vi) in the presence of CDs/TiO_2_ mesocrystal composite. Adapted with permission from ref. 125. Copyright 2018 Elsevier. (b) Proposed photo-Fenton synergistic mechanism of nitrogen-doped GO/Fe_2_O_3_ mesocrystal nanocomposites. Adapted with permission from ref. 130. Copyright 2017 Elsevier. (c) Possible photocatalytic mechanism of carbon-modified NaTaO_3_ mesocrystals. Adapted with permission from ref. 131. Copyright 2014 Royal Society of Chemistry.

CDs with a large number of active groups (including hydroxyl groups and carboxyl groups) have been widely applied in photocatalysis due to their excellent photoelectric properties, excellent water solubility, and suitable chemical reactivity. Hence, it is expected that the coupling of CDs with mesocrystals could inhibit photogenerated charge recombinations, leading to enriched photoelectrons. Bian and coworkers have demonstrated the enhanced photoreduction of Cr(vi) to Cr(iii) by using CDs-coupled TiO_2_ mesocrystals, where CDs played the roles of electron collectors and active sites (Fig. 22a). Furthermore, the CDs coupled on TiO_2_ mesocrystals can facilitate photogenerated charge separation. In addition, the authors have found that the positive charges on the surface of CDs/TiO_2_ was in favor of the selective adsorption of Cr(vi) and rapid desorption of Cr(iii), which obviously promotes the photocatalytic reduction of Cr(vi) and retention of photoreduction activity. Therefore, the photoreduction of Cr(vi) performance of the as-prepared CDs/TiO_2_ is about 5.4 times higher than that of pure TiO_2_ mesocrystals under UV-light illumination.^117^ Yan and coworkers have reported that CDs/TiO_2_ mesocrystal composites synthesized through a simple one-pot solvothermal method can exhibit effective photocatalytic activity in the degradation of methyl orange under visible-light irradiation.^126^

As an sp2-bonded carbon sheet with the thickness of a single atom, graphene has received much more attention owing to its large surface area, good flexibility, high electrical conductivity, and high chemical stability, which allows it to be an effective host support for the heterogeneous growth of the desired active guest materials because the surface functional groups, such as hydroxyl groups, act as favorable nucleation sites for guest materials.^127^ Moreover, graphene can decrease the recombinations of photogenerated electron–hole pairs, increasing the charge transfer rate of electrons and surface-adsorbed amount of chemical molecules through π–π interactions. Consequently, the integration of graphene and mesocrystal semiconductors is promising in the field of photocatalysis. For example, Yang and coworkers have employed a facile template-free solvothermal method for obtaining well-dispersed spindle-like anatase TiO_2_ mesocrystals anchored onto graphene nanosheets, which were synthesized by mixing GO with acetic acid under ultrasonication, followed by the dropwise addition of tetrabutyl titanate into the above suspension.^128^ The as-synthesized graphene/anatase TiO_2_ mesocrystals can considerably enhance the photocatalytic activity of TiO_2_ under visible-light irradiation.

As a graphene derivative with an edge-bearing oxygen functionality, GO provides the advantage of uniformly loading metal oxide nanoparticles on its surface through oxygen-containing groups as nucleation centers.^129^ Nonetheless, the poor electron migration ability of GO is undesirable in photocatalysis, which limits the charge transfer between the nanoparticles and GO. Decreasing the oxygen-containing groups was an effective approach to increase the charge transportation capacity of GO. Liu and coworkers have synthesized a pyrrolic nitrogen (N)-doped GO/Fe_2_O_3_ mesocrystal nanocomposite by a simple solvothermal route and optimizing the oxygen-containing groups on GO. The as-prepared N-doped GO/Fe_2_O_3_ mesocrystals can enhance the efficient separation of electron–hole pairs and promote the fast conversion of Fe(ii)and Fe(iii) in photo-Fenton synergistic reactions because of the excellent electroconductivity of pyrrolic-N-doped GO and a large specific surface area (Fig. 22b).^130^ The photodegradation rate of methyl blue increased by 1.5 times and the conversion rate of glyphosate increased by 2.3 times for the GO/Fe_2_O_3_ mesocrystals under visible-light irradiation as compared to bare Fe_2_O_3_ mesocrystals.

Besides the above CDs, GO, and graphene, amorphous carbon can be also used to improve the photocatalytic activity of mesocrystal photocatalysts. Wu and coworkers have found that carbon-modified NaTaO_3_ mesocrystal nanoparticles can be prepared by a one-step solvothermal method using TaCl_5_ and NaOH as the starting materials and distilled water/ethylene glycol mixed solution as the reaction solvent in the presence of appropriate amounts of glucose.^131^ These carbon-modified NaTaO_3_ mesocrystals exhibited excellent efficiency for continuous NO gas conversion under UV irradiation, short-wavelength visible light (>400 nm), and even long-wavelength visible light (>510 nm), which was considerably better than those of unmodified NaTaO_3_ and commercial P25 due to the large specific surface area, high crystallinity, and carbon-induced visible-light harvesting (Fig. 22c).^131^

The rapid emergence of novel carbon–mesocrystal heterogeneous nanostructures can provide a new opportunity to further understand the fundamental importance of mesocrystals as well as to improve their practical applicability. Notably, other carbon nanomaterials possessing unique physicochemical properties, such as carbon nanotubes (CNTs), C_60_, polymer polypyrrole (PPy), and metal–organic frameworks (MOFs), should be decorated with mesocrystals in the future.

### 3.2 Doping

It is well known that doping is a well-demonstrated strategy for effectively enhancing the physicochemical properties by changing the electronic structure of semiconductors; therefore, studies on doped mesocrystals have attracted increased attention. Herein, we will discuss structure-related photocatalytic mechanisms based on some typical doped-mesocrystal photocatalyst examples. Table 2 summarizes the metal- and non-metal-doped mesocrystal photocatalysts and their physiochemical properties, as well as their photocatalytic applications.

**Table 2:** Doped mesocrystal photocatalysts and their properties

Doping element	Precursor	Synthesis method	Application	Ref.
Sr^2+^	NaTaO_3_ mesocrystals	Hydrothermal method	Electrons exciting	134
Sr^2+^	NaTaO_3_ mesocrystals	Molten salt method	Photocatalytic hydrogen generation	135
Ti^3+^	TiO_2_ mesocrystals	Solvothermal synthesis	Photocatalytic removal of NO gas	137
Ti^3+^	Au/Cl–TiO_2_ mesocrystals	Vapor hydrolysis + photoreduction	Photocatalytic hydrogen generation	138
Cr^3+^ and Sb^5+^	TiO_2_ mesocrystals	Hydrothermal method	Photodegradation of methyl orange	139
Nb^5+^ and Sb^5+^	TiO_2_ mesocrystals	Microwave-assisted approach	Photodegradation of methylene blue and rhodamine B	140
Zn^2+^	Fe_3_O_4_ mesocrystals	Solvothermal synthesis	Photo-Fenton	141
F	TiO_2_ mesocrystals	Topotactic transformation	Photocatalytic hydrogen generation	145
N and F	TiO_2_ mesocrystals	Topotactic transformation	Photodegradation of methylene blue	149
N and F	TiO2 mesocrystals	Hydrothermal method	Photodegradation of 4-nitrophenol and rhodamine B	150

#### 3.2.1 Metal doping

Generally, the introduction of metallic elements would intensify additional binding functions, which induces unique photocatalytic properties in the doped system by decreasing the bandgap and increasing the absorption of visible light.^132,133^ In order to import metal ions into the framework of mesocrystals, the corresponding soluble salt is always uniformly mixed with the mesocrystal precursors; therefore, metallic impurities get simultaneously doped into the mesocrystals.

**Fig. 23:**
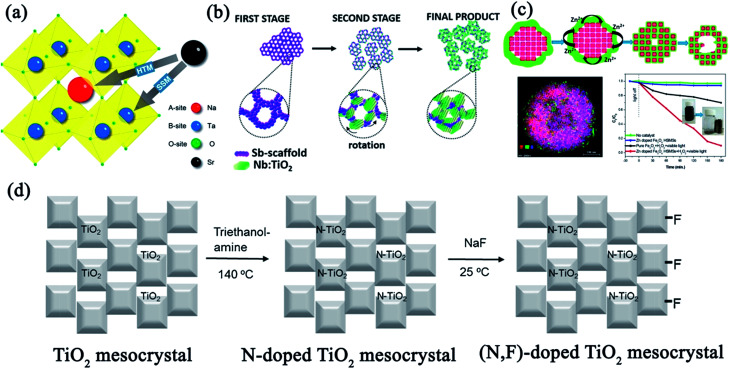
(a) Crystal structure of NaTaO_3_–SrSr_1/3_Ta_2/3_O_3_ solid solutions. Adapted with permission from ref. 134. Copyright 2015 American Chemical Society. (b) Schematic of the formation mechanism of Sb-*meso*Nb/TiO_2_. Adapted with permission from ref. 140. Copyright 2017 American Chemical Society. (c) Schematic of the growth process for Zn-doped Fe_3_O_4_ hollow sub-microsphere mesocrystals and their photocatalytic activities. Adapted with permission from ref. 141. Copyright 2017 American Chemical Society. (d) Schematic illustration of a facile hydrothermal treatment synthesis process of N-doped TiO_2_ mesocrystals and (N,F)-doped TiO_2_ mesocrystals. Adapted with permission from ref. 150. Copyright 2016 Elsevier.

An and coworkers have synthesized Sr^2+^-doped NaTaO_3_ photocatalysts by solid-state and hydrothermal routes.^134^ The as-synthesized NaTaO_3_–SrSr_1/3_Ta_2/3_O_3_ solid solution was a Sr-rich shell covered with a Sr-poor core. This heteroepitaxial core–shell interface can induce surface reconstruction with regularly separated steps due to lattice mismatch. Therefore, the recombination of electron–hole pairs was limited in solid-solution photocatalysts. The steady-state photogenerated electrons increased by up to 180 times in the solid-solution photocatalysts under Hg–Xe lamp irradiation, in which both A sites and B sites of the perovskite-structured lattice were doped with Sr^2+^ (Fig. 23a). Surface reconstruction and enhanced electron population were absent in the A-site-doped photocatalysts.^134^ In addition, a simple molten salt route without using any organic additives has been employed to prepare Sr^2+^-doped NaTaO_3_ mesocrystals with high crystallinity and orientation-aggregated morphology, which exhibited excellent photocatalytic hydrogen generation activity because of their nanosteps, high porosity, and preferred oriented direction.^135^

As for TiO_2_ photocatalysts, Ti^3+^ self-doping can result in the visible-light response of TiO_2_ materials without introducing recombination centers for the photogenerated charges.^136^ Therefore, the achievement of Ti^3+^ self-doped anatase TiO_2_ mesocrystals would be significant for the construction of an ideal photocatalyst. For instance, Zhao and coworkers have prepared spindle-like Ti^3+^ self-doped anatase TiO_2_ mesocrystals through the calcination of pre-synthesized anatase TiO_2_ mesocrystals in N_2_ atmosphere.^49^ However, volatile organic solvents and an expensive surfactant additive were inevitably utilized in this work. Hence, it is highly desirable to develop a simple, green synthesis strategy for fabricating Ti^3+^-doped TiO_2_ mesocrystals. Recently, Tan and coworkers have reported a novel method for the growth of spindle-shaped anatase TiO_2_ mesocrystals through the one-step hydrolysis reaction of TiCl_3_ in the green and recyclable media, polyethylene glycol. Subsequently, Ti^3+^ sites can be easily generated in the anatase crystal lattice, leading to the formation of Ti^3+^ self-doped mesocrystals after being annealed in vacuum. These as-transformed Ti^3+^ self-doped anatase mesocrystals exhibited enhanced visible-light photocatalytic activity toward the removal of NO gas, which could be attributed to their intrinsic Ti^3+^ self-doping nature, as well as high crystallinity and high porosity of the mesocrystalline architecture.^137^ Moreover, Yu and coworkers have successfully prepared willow-leaf-like plasmonic Ti^3+^-doped Au/Cl–TiO_2_ mesocrystals by means of modified two-phase vapor hydrolysis and photoreduction methods. The as-prepared Ti^3+^-doped Au/Cl–TiO_2_ mesocrystals displayed higher visible-light harvesting and visible-light photocatalytic activity for hydrogen evolution, which was 208.70 times as high as that of P25 TiO_2_ and 1.59 times as high as that of Ti^3+^-doped TiO_2_ mesocrystals.^138^ Therefore, the co-doping of TiO_2_ mesocrystals might provide remarkably higher activity than that of single doping and bare TiO_2_. Mesocrystalline Cr- and Sb-co-doped anatase TiO_2_ nanoparticles with a solid solution characteristic were prepared by the hydrothermal aging of an aqueous solution containing titanium alkoxide, chromium acetylacetonate, antimony acetate, and triethanolamine as the stabilizer, which could improve the visible-light harvesting ability, thereby revealing high efficiency in the degradation of methyl orange dye under visible-light irradiation.^139^

Multicomponent Sb–Nb:TiO_2_ mesocrystals have been synthesized by a microwave-assisted nonaqueous sol–gel method, with size of 25–35 nm and composed of crystallographically aligned Nb:TiO_2_ subunits, embedded in a porous amorphous Sb-rich scaffold. The formation of Sb–Nb:TiO_2_ mesocrystals is responsible for a particle-based assembly mechanism. In this process, the Sb scaffold acted as a nucleation site for the construction of Nb:TiO_2_ subunits, which grew and rotated in a mutual crystallographic orientation. It then prevented the complete fusion of Nb:TiO_2_ subunits, leading to the porosity of Sb–Nb:TiO_2_ mesocrystals (Fig. 23b).^140^ When compared with undoped TiO_2_, Sb–Nb:TiO_2_ mesocrystals exhibited superior photocatalytic activity for the degradation of organic dyes under simulated solar or visible light, which can be attributed to the high crystallinity, abundant porosity, and additional exposed reactive surfaces.

In addition, magnetic recyclable mesocrystalline Zn-doped Fe_3_O_4_ hollow sub-microspheres were successfully prepared through a facile one-step solvothermal method and were used for fabricating efficient heterogeneous photo-Fenton catalysts.^141^ A possible growth mechanism of doped mesocrystalline hollow materials was proposed. Initially, Fe_3_O_4_ mesocrystals were assembled by oriented nanocrystals, and Zn-rich amorphous shells grew onto the surfaces. Subsequently, Zn element gradually diffused into Fe_3_O_4_ mesocrystals to generate Zn-doped Fe_3_O_4_ because of the Kirkendall effect by increasing the reaction time, in which a directional flow of zinc species at the Fe_3_O_4_ interfaces could result in the formation of voids in the products. Simultaneously, the inner solids would be dissolved, and the outer particles would grow larger due to the Ostwald ripening process, finally resulting in the formation of a hollow structure with a porous shell. The Zn-doped hollow Fe_3_O_4_ mesocrystals possessed high and stable photo-Fenton activity for the degradation of rhodamine B and cephalexin under visible-light irradiation in the presence of H_2_O_2_ (Fig. 23c),^141^ and it could be easily separated and reused by an external magnetic field.

In brief, metal ions were extensively employed as dopants for mesocrystal photocatalysts. Generally, the introduction of metal ions can form new energy levels in the bandgap, optimize the visible-light response, and accelerate the separation of the electron–hole charges. Although many studies on transition metal-doped mesocrystals have been reported, non-metal doping is still in its infancy.

#### 3.2.2 Non-metal doping

Thus far, non-metal-doped mesocrystals have mainly focused on TiO_2_ mesocrystals. Previously, it has been confirmed that the substitution of an oxygen atom with a fluorine (F) atom can effectively improve the photocatalytic activity of TiO_2_.^142–144^ However, obtaining n-type F-doping in TiO_2_ mesocrystals was still a challenge, until Majima and coworkers reported that an *in situ* F-doping of TiO_2_ superstructures can be used for efficient visible-light-driven hydrogen generation. In this case, the details of crystal growth and dynamic structure evolution during the topotactic transformation from the initial intermediate NH_4_TiOF_3_ to HTiOF_3_, TiOF_2_, and F-doped TiO_2_ mesocrystals were revealed.^145^ Further, F-doped TiO_2_ mesocrystals synthesized at 500 °C can accelerate the electronic mobility for efficient visible-light-driven hydrogen production.

It has been demonstrated that N-doping is one of the most efficient avenues to generate N 2p in the localized mid-gap state, resulting in an increase in the thermal stability and decrease in recombination centers.^146–148^ Therefore, it is necessary to integrate N- and F-doping into TiO_2_ mesocrystals, which could combine the advantages of these single dopants for further optimizing the photocatalytic activity. With regard to synthesizing co-doped mesocrystals, only TiO_2_ mesocrystals with N- and F-co-doping have been studied thus far. Topochemical transformation is an effective method for the synthesis of (N,F)-co-doped TiO_2_ (NFT) mesocrystals exposed with (001) facets. The distribution of two dopants in NFT strongly depended on the annealing temperature. It was found that NFT (500 °C) displayed the highest photocatalytic efficiency from Cr(vi) to Cr(iii) because of the highest concentration of N with the surface modification from F coupling.^149^ Similarly, Majima and coworkers have found that N-doped TiO_2_ mesocrystals can be synthesized by a facile hydrothermal treatment with triethanolamine, while F-doped TiO_2_ mesocrystals were easily formed by a simple stirring of TiO_2_ mesocrystals with NaF at room temperature. Therefore, (N,F)-co-doped TiO_2_ mesocrystals were prepared by a two-step post-modification process. A schematic illustration of the preparation of N-doped and (N,F)-co-doped TiO_2_ mesocrystals is shown in Fig. 23d.^150^ In this system, it was seen that doping has no effect on the crystal structure of TiO_2_ mesocrystals, and the plate-like structure and high surface area can be retained. These (N,F)-co-doped TiO_2_ mesocrystals displayed high photocatalytic activities for the degradation of rhodamine B and 4-nitrophenol under visible-light irradiation, which was attributed to the synergistic effect of N- and F-doping. The introduction of heterogeneous N atoms enhanced visible-light absorption, resulting in a decrease in the bandgap energy by adjusting the concentration of N, while F increased the production of hydroxyl radicals, adsorption, and charge separation efficiency. Therefore, TiO_2_ mesocrystals with N- and F-co-doping provided a particular interest in promising visible-light-driven photocatalytic efficiency and allowed us to devote the effect on developing new materials as a photocatalyst.

## 4. Conclusions and perspective

Mesocrystal photocatalysts with tunable crystalline phases, sizes, and morphologies have been widely studied owing to crystallographically highly ordered superstructures for photogenerated charge transfer and separation, high surface area, and ordered porosity as compared to corresponding single crystal and polycrystalline nanomaterials. Herein, a comprehensive review on the synthesis strategies and growth mechanisms, morphological diversities, functional modifications including hybridization and doping, and current applications, as well as the corresponding structure-related photocatalytic mechanisms, have been summarized for different types of mesocrystal photocatalysts, such as TiO_2_ (anatase), TiO_2_ (rutile), ZnO, CuO, Ta_2_O_5_, BiVO_4_, BaZrO_3_, SrTiO_3_, NaTaO_3_, Nb_3_O_7_(OH), In_2_O_3−*x*_(OH)_*y*_, and AgIn(WO_4_)_2_. Recent advances in designated tailoring of various kinds of mesocrystals provides a promising approach for the rational design and construction of high-performance photocatalysts, as well as the enrichment of their surface atomic/or electronic and interfacial properties. Although extensive investigations and significant achievements made during the past decades, as discussed in this review article, there is still a long way ahead before mesocrystal photocatalysts can be comprehensively understood and satisfactorily used in industrialization. In order to achieve this, we have numerous tasks to particularly investigate in the future.

With regard to the synthesis of novel mesocrystal photocatalysts, an important task is to promote the synthesis of mesostructural architecture with desired crystallographic facets, particularly high-index facets. The selection of suitable capping agents for constructing targeted mesostructures based on computer-assisted surface energy variations might considerably promote the synthesis of targeted mesocrystalline photocatalysts. With regard to popular TiO_2_ mesocrystals, the literature mainly focuses on the anatase phase with diverse architectures, whereas studies involving the rutile phase are still in their infancy. Moreover, the size-controlled synthesis of polyhedral TiO_2_, particularly ultrathin monodisperse nanoparticles (size < 100 nm), is an urgent task. Notably, the as-reported polyhedral TiO_2_ mesocrystal photocatalysts generally exhibited low-index (001) and (101) facets, which strongly restrained investigations on facet-dependent properties. Therefore, considerable efforts should be further expended to explore the synthesis of novel polyhedral TiO_2_ mesocrystals exposed with controllable-index facets for uncovering the relationship between surface structures and performances.

Apart from TiO_2_, it is urgently necessary to focus more attention on enriching the structural and morphogenetic aspects, as well as formation mechanisms, of other mesocrystals, which are beneficial toward tuning the size, morphology, crystalline phase, monodispersity, surface atomic arrangement, and intrinsic electronic structure. Significantly, the surfactant-free, large-scale controllable synthesis of low-dimensional mesocrystalline architectures is still a challenge for all the current mesocrystal species because the general growth mechanism is still uncertain.

In order to optimize the performances of mesocrystal photocatalysts, functional modifications, including doping and hybridization, should be thoroughly explored. For the doped mesocrystal photocatalysts, the current literature concentrates mainly on metal doping, studies have not focused on morphology- or sized-controlled syntheses of doped ones. Moreover, non-metal doping should also be taken into consideration because it is a promising research avenue. In addition, the co-doping and selective doping of heteroatoms for synergistically optimizing the electronic structures is still a formidable challenge. As a result, the development of doped precursors for annealing or transformation might be an effective strategy to achieve the controllable synthesis of doped mesocrystals. For mesocrystal-based hybridized photocatalysts, decorating active components with controllable structures on specific sites of mesocrystals should attract extensive attention, and revealing the relationship between facet-dependent interfacial and photocatalytic activities is still a primary target. Significantly, it is imperative to focus more efforts toward the synthesis of multicomponent mesocrystals (including heterojunction and hetero-phase junctions) for exploring novel photocatalysts. Notably, the theoretical calculations involving the electronic structures of doped mesocrystals and interfacial structures of hybridization architectures should be investigated for the actual cognition of photocatalytic mechanisms.

To summarize, the comprehensive and in-depth review on the synthesis engineering and functional modifications (including doping and hybridization), as well as their corresponding structure-related enhanced photocatalytic mechanisms, can facilitate the development of new mesocrystal-based photocatalysts.

## Conflicts of interest

There are no conflicts to declare.

## Supplementary Material
